# Overview of basic design recommendations for user-centered explanation interfaces for AI-based clinical decision support systems: A scoping review

**DOI:** 10.1177/20552076241308298

**Published:** 2025-01-23

**Authors:** Ian-C. Jung, Katharina Schuler, Maria Zerlik, Sophia Grummt, Martin Sedlmayr, Brita Sedlmayr

**Affiliations:** 1 Institute for Medical Informatics and Biometry, Faculty of Medicine and University Hospital Carl Gustav Carus, TUD Dresden University of Technology, Dresden, Germany

**Keywords:** scoping review (ScR), explainable artificial intelligence (XAI), explanation user interface (XUI), user-centered design, clinical decision support systems, medical informatics

## Abstract

**Objective:**

The application of artificial intelligence (AI)-based clinical decision support systems (CDSS) in the healthcare domain is still limited. End-users’ difficulty understanding how the outputs of opaque black AI models are generated contributes to this. It is still unknown which explanations are best presented to end users and how to design the interfaces they are presented in (explanation user interface, XUI). This article aims to provide an overview of recommendations for the user-centered design of XUIs from the scientific literature.

**Methods:**

A scoping review was conducted to identify recommendations for the design of XUIs. Articles published between 2017 and 2022 in English or German, presenting original research or literature reviews, focusing on XUIs for end users or domain experts, which are intended for presentation in graphical user interfaces and from which recommendations could be extracted were included in the review. Articles were retrieved from Scopus, Web of Science, IEEE Explore, PubMed, ACM Digital Library, and PsychInfo. A mind map was created to organize and summarize the identified recommendations.

**Results:**

From the 47 included articles, 240 recommendations for the user-centered design were extracted. The organization in a mind map resulted in 64 summarized recommendations.

**Conclusion:**

This review provides a synopsis of basic recommendations for the user-centered design of XUIs, focusing on the healthcare domain. During the analysis of the articles, it became clear that no specific and directly implementable design recommendations for AI-based CDSS can be given, but only basic recommendations for raising awareness about the user-centered design of XUIs.

## Introduction

In the healthcare domain, research in artificial intelligence (AI) started slowly^1^ but has since then seen a substantial increase in research activity.^1–4^ This increase is assumed to continue in the future.^1^ The application of AI in the healthcare domain is expected to have a transformative impact on the healthcare domain.^3,5^ The anticipated benefits of AI in the healthcare domain are manifold, for example, improved consultations, health assistance, medication management, diagnostics, precision medicine,^3^ or support in clinical decision-making.^4^ Promising results have been shown, for example, in radiology, where several commercially available applications exist, but even in this advanced sector, the application in the clinical working process is limited.^6,7^ Incorporating promising AI methods into clinical practice has not been the norm; only a few applications have been able to cross this gap.^5^ It is a frequently formulated aspiration for the future that the application of AI-based systems will lead to a partnership between the healthcare providers and AI-based systems rather than a replacement of the healthcare providers.^1,3,5,8,9^ To fully realize the expected transformative impact of AI-based systems in healthcare and deliver the expected benefits, a number of problems and challenges need to be addressed. The healthcare providers’ acceptance of AI-based systems and the related topics of trust and understandability are identified problems when it comes to AI-based systems^8,10–12.^ These are of utmost importance for the adoption of AI-based systems in healthcare, both nationally and internationally, as the decisions made in healthcare can have life-changing consequences. A lack of transparency could lead to skepticism, hesitation, rejection, or, conversely, unwarranted trust in the systems, especially for AI-based clinical decision support systems (CDSS). Artificial intelligence-based CDSS is one form of AI-based systems in the healthcare domain. For the remainder of the paper, the term CDSS is defined as an application or system that assists medical professionals or patients in the medical decision-making process in any form. Artificial intelligence-based CDSS differ from rule- or guideline-based CDSS, where the outputs are generated based on consolidated clinical knowledge, for which the CDSS are built as a manifestation of this existing knowledge base. In contrast, for an AI-based CDSS, the knowledge base is an AI model trained on a large amount of data that is designed to operate without the definition of rules based on consolidated clinical guidelines. These points are important against the backdrop of opaque black models for which it is not directly clear why a output was generated and hopes for a partnership between human healthcare providers and AI-based systems. Explainable AI (XAI) is a method that tries to open these black boxes and explaines the outputs of AI models. Explainable AI is seen as a way to overcome the limitations, and its importance for the medical field has been emphasized.^3,5,13–18^ As a result, the interest in XAI in the healthcare domain has grown rapidly in recent years.^19^

The terminology used in the field of XAI is complex and a topic in itself,^20–26^ which is beyond the scope of this review. For the sake of simplicity in this article, an explanation for an AI-based system is considered to be any information that makes it easier for the user to make sense of or understand the system's output or to facilitate its appropriate use. The interfaces in which these explanations are presented to users can be referred to as explanation user interface (XUI).^27^ In this scoping review, the term XUI will entail the explanations presented to the user and the interface in which these are presented, if not stated otherwise. It has been advocated for a user-centered design of XUIs in the healthcare domain to ensure an effective and intuitive use of AI-based systems to advance the useful application in the clinical working processes.^12,28,29^

### Related work

A few broad reviews exist that touch on the user-centered design of XUIs. For example, Mohseni, Zarei, and Ragan^25^ reviewed the computer science literature for articles regarding the topic of XAI, focusing on XAI's user-centered design goals and evaluation methods. A systematic review by Nunes and Jannach^30^ provides a broad overview of explanations for advice-giving systems, focusing on the content and presentation of explanations, the generation methods for the explanations, elevations methods, and results of these evaluations. Other review articles focus on specific aspects of the user-centered design of XUIs, for example, evaluations,^31–33^ interaction with the users,^27,34^ or potential cognitive biases when dealing with XUIs.^35^ In addition, a few reviews focus exclusively on XAI for the healthcare domain. With a focus on technical aspects, some authors provide an overview of XAI methods^15,19,36,37^ or the selection of XAI methods and their evaluation.^17^ Others present human–computer interaction challenges and research gaps regarding XAI in the healthcare domain in general^38^ or regarding the subfield of CDSS^39^ or catalog the explanation needs of users for AI-based diagnostic systems.^40^ There appears to be a gap in the literature that provides a synopsis of recommendations for the user-centered design of XUIs based on the recent scientific literature, which facilitates the application of the recommendations in the medical domain.^18^

### Rational and research question

This scoping review will be conducted to fill this gap. By examining the emerging research results, the scoping review will collect, organize, and present recommendations for the user-centered design of XUIs in a summarized manner. The review will highlight the recommendations based on literature focusing on CDSS, as it will be one main application area for XUIs. This review is intended to sensitize researchers and programmers who are designing XUIs for AI-based CDSS to the complexity of design decisions that should be made mindfully when designing XUIs for AI-based CDSS. The recommendations will be helpful for projects in which XUIs for AI-based CDSS have to be designed with limited expertise on the topic. The targeted users of the collected design recommendations are researchers and developers of the XUIs for AI-based CDSS. The scoping review can serve as a way to sensitize this audience to the topic and provide starting points for design decisions that have to be made to design helpful XUIs. In addition, the review will gather information, allowing the reader to independently assess the appropriateness of the recommendations for a specific use case.

Although the topic of XAI in the healthcare domain is gaining attention, the number of articles included in review articles with a specific focus on XAI in the healthcare domain is often limited.^36,39^ To combat the limited amount of available literature on the design of XUIs in the healthcare domain, especially for AI-based CDSS, and to ensure the consideration of the highest possible number of design recommendations and aspects relevant to the design of XUIs, this scoping review is performed in two different layers. The first layer focuses on the design of XUIs without a restriction to AI-based CDSS. In contrast, the second layer focuses specifically on the design of XUIs for AI-based CDSS. To achieve this, the scoping review will investigate the following research questions:

1. What recommendations exist for a user-centered design of explanations or XUIs for AI-based systems in general?
2. What recommendations exist for the user-centered design of explanations and XUIs for AI-based decision support systems in the medical domain?

## Methods

The authors planned and performed a scoping review to investigate the research questions. The planning, execution, and reporting of the review were adapted according to published best practices for scoping reviews^41^ and the Prisma ScR reporting guideline^42^ (see Appendix A for the “Prisma ScR Reporting Checklist”).

### Scoping review protocol and screener handout

The first author planned and documented the scoping review within a scoping review protocol (Appendix B); this protocol was not published but known to the authors of this review. In addition, a handout (Appendix C) for each step of the source selection process was provided to all researchers participating in these steps. The handout contained the relevant information for performing the step in question.

### Eligibility criteria

During the source selection process, the researchers evaluated the articles based on the eligibility criteria presented in Table 1. The researcher had access to a more extensive version of the eligibility criteria for clarity of the decisions; that is, available in the digital appendix in the handout (Appendix C) and review protocol (Appendix B).

**Table 1.** Short version of inclusion and exclusion criteria used during the source selection process of the scoping review.

Phase	Inclusion	Exclusion
Title Abstract Full-Text Scan	Articles published from 2017 to 2022	Articles published before 2017
	Language: English or German	Language: Not English or not German
	Article Type: • Original Research • Literature Review	Article Type: • Not Original Research • Not Literature Review • Scientific Thesis
	Article focus: • User-centered design of XUIs for AI-based systems	Article focus: • Backend aspects of XUIs (e.g., algorithmic or technical focus) • Philosophical, legal, or ethical aspects of XAI.
	Recipients of XUIs: • Domain Experts • End-Users	Recipients of XUIs: AI Experts (e.g., AI researchers, data scientists, programmers, etc.)
	XUIs are intended to be presented as a graphical user interface.	XUIs are not intended to be presented as a graphical user interface.XUIs are intended for autonomous systems.
Full-Text Scan	The researchers have full-text access to the article.	The researchers have **no** full-text access to the article.
	Recommendations are extractable from the description in the article.	Recommendations are **not** extractable from the description in the article.

### Information sources

The databases for the article search were selected to cover various research areas, as the topic of user-centered XAI appears interdisciplinary. The Following databases were queried on 2022/02/13: Scopus, Web of Science, IEEE Explore, PubMed, ACM Digital Library, and PsychInfo.

### Search

The databases were queried with the following search string:

(Explainab* OR “Explanation” OR Transparen* OR Interpretab* OR Understandab* OR Justification) AND (AI OR “Artificial Intelligence” OR “Machine Learning” OR ML OR “Intelligent System” OR “Intelligent Agent” OR “Recommender System”) AND (“Human-Centered” OR “User-Centered” OR “Usability”)

The query searched the title, abstract, and keywords fields. The search string was adjusted to the database-specific syntaxes; each adjusted search string can be found in the review protocol. All search fields were searched for the “IEEE Explore” database since a transformation to limit the search fields to title, abstract, and keywords was impossible without changing the search string altogether.

### Source selection process

The source selection process consisted of a duplicate removal, a title abstract scan, and a full-text scan. For each phase, the first author created a coding sheet for the reasons for exclusion (Appendix D). For each phase, a calibration process was performed until the research team members were confident that a mutual understanding was reached. At least two researchers assessed each article for inclusion or exclusion during the title-abstract and full-text screening. In case of disagreements between the researchers, they had the opportunity to resolve them in a bilateral meeting. If no consensus was reached, a third independent decision was obtained from a research team member. The majority decision was followed.

### Data chattering process and data items

The first author created an initial draft of the data extraction table, which was discussed in multiple team meetings. Before the data chartering process started, a calibration phase was performed to generate a mutual understanding of the data items. Two researchers independently extracted the data for each article during the data chartering process. The first author combined the data extractions for all articles in a table. Each researcher involved in the data chartering process checked for each of their extractions whether they approved the merged extractions or if changes were necessary. Disagreements between the researchers were resolved in bilateral meetings.

The extracted data items can be obtained from the data extraction table (Appendix E) in the digital appendix. Data for 18 data items were extracted: information for identification of the article; the aim or purpose of the article; the domain in which the XUI was used/designed (e.g., finance), the user role which uses the XUI (e.g., social worker), and the method(s) used to generate the information on which the recommendations are based; information describing an evaluation of the XUI, if an evaluation was performed; information describing the AI method for which the XUI was created in the articles. Furthermore, recommendations for the user-centered design of XUIs were extracted. For each recommendation, the relevant text passage was extracted. If the relevant text passage was not phrased as a recommendation, the authors of this review phrased a short recommendation for the relevant text passage. In the data extraction table, it was noted whether recommendations were deduced (“#D”) based on the information provided in the original article or whether the recommendations were directly provided in the original article (“#P”).

### Critical appraisal

The researchers conducted a critical appraisal (CA) of each article using a customized CA tool. This tool was created after assessing common CA tools and reporting guidelines, ensuring it could accommodate the wide variety of methodologies anticipated while remaining practical. The CA tool and the appraisal results are available in the appendix (Appendix F). These were not intended to alternate the inclusion or exclusion decisions for the articles or the assessment of the recommendations; rather the CA aimed to enable the authors of this review to assess the quality of the sources.

### Synthesis of results

**Figure 1.**
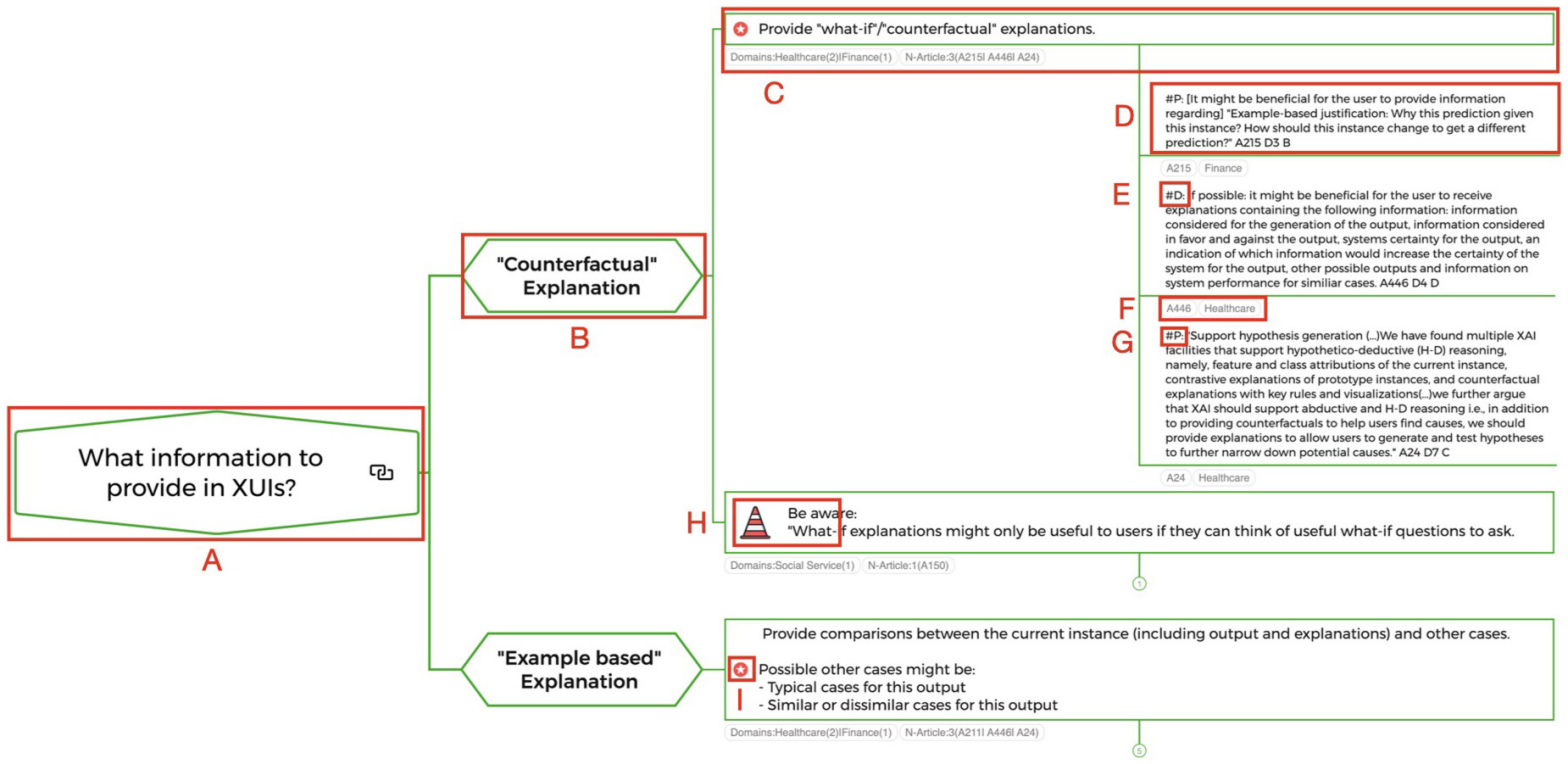
Mind map excerpt with highlighted structural aspects: (A) Main question; (B) Orientation node; (C) Summarized recommendation; (D) Original extracted recommendation; (E and G) Identification of recommendation origin, “#P” = provided in the original article, “#D” = deduced by the authors of this scoping review; (F) Information about source article; (H) Highlight of recommendations which inform of possible pitfalls; (I) Highlight of summarized recommendation based on article from the healthcare domain.

After all, researchers involved in the data extraction had reviewed the summarized data extraction, the first author structured the extracted recommendations into a mind map based on their content. The first layer of the mind map was structured by questions that someone who would be designing an XUI might ask. For each identified question, a mind map branch with an individual substructure was created based on the content of the summarized recommendations. In each branch, the main question (e.g., A in Figure 1) is followed by a number of nodes with keywords for a better orientation (e.g., B in Figure 1). The summarized recommendation is presented after an individual sequence of orientation nodes (e.g., C in Figure 1). The last layer of the branch contains the original extracted recommendations as leaves of the branch (e.g., D in Figure 1).

For each summarized recommendation, it is reported in the mind map how many articles the summarized recommendation represents and to what domain these articles belong (e.g., C in Figure 1). In the last layer of the mind map, IDs were included to disclose to which article the extracted recommendation belongs, for example, “A110” (e.g., F in Figure 1). The IDs can be matched to the individual data extractions in the data extraction table. The domain of the article extended this information. In the last layer of the mind map, “#D” or “#P” indicates whether the recommendation was deduced from the information in the article or provided directly in the original article (e.g., E or G in Figure 1). Each summarized recommendation based on at least one article from the Healthcare domain is marked in the mind map with a red star icon (e.g., I, in Figure 1).

The recommendations were rearranged and grouped until a stable mind map emerged. The first author summarized the recommendations for each group of recommendations. If some recommendations were contradictory or deviating, a broad wording was chosen to reflect this accurately. The researchers involved in the data extraction reviewed and proposed changes to the mind map in two rounds of feedback.

For the analysis of the second research question, only recommendations were considered which were based on research articles focusing on AI-based CDSS. 

During the synthesis of the results and the analysis of the data extraction, R Version 4.3.1 with tidyvers^43^ was used, and the mind map was created using the software xmind Version 23.11.04336.

## Results

**Figure 2.**
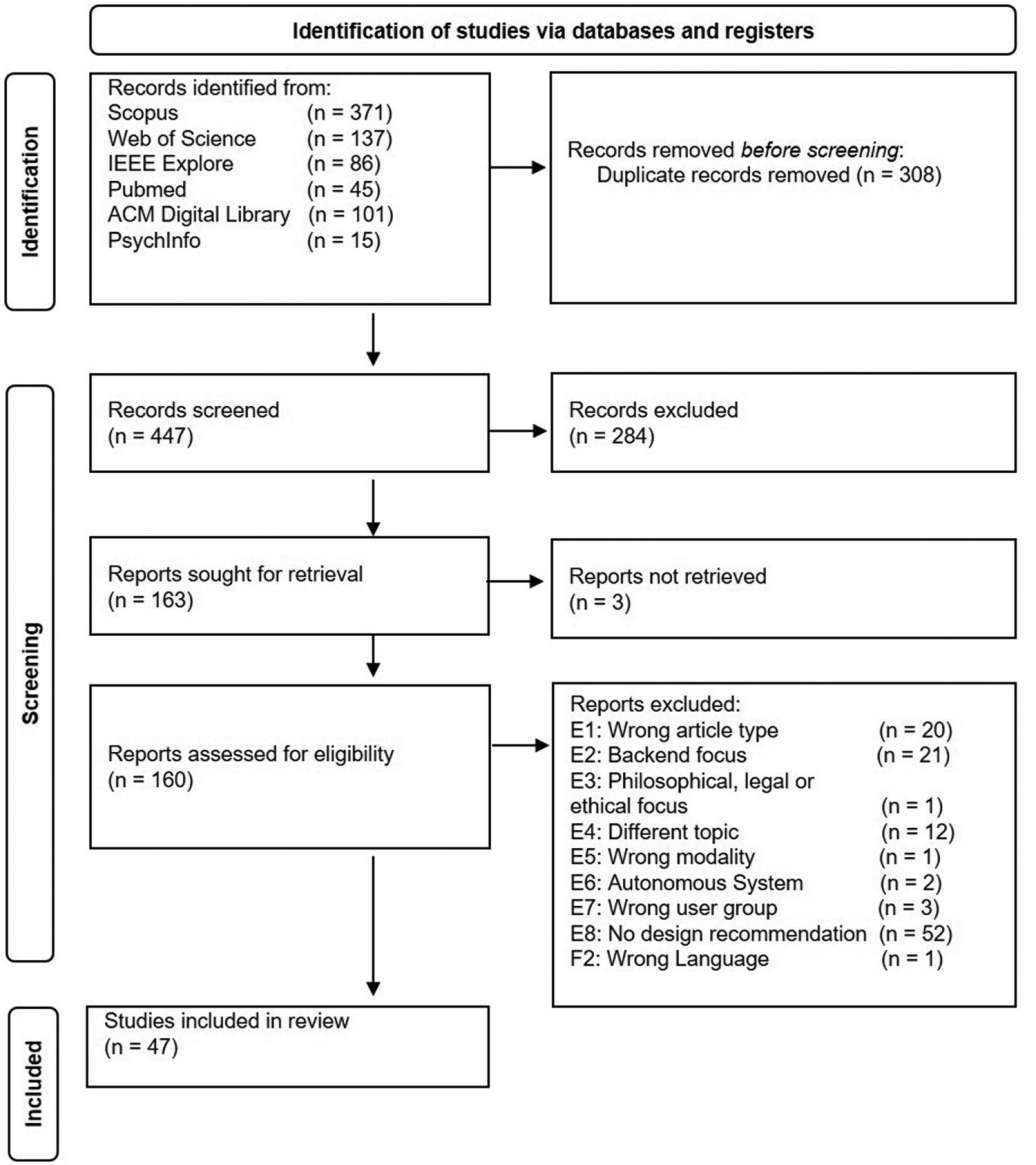
Flowchart describing the source selection process.

The database queries resulted in 755 retrieved articles. After removing 308 duplicates, 447 articles were screened for inclusion during the source selection process, resulting in 47 articles in the scoping review. Figure 2 presents a flowchart based on Page et al.^44^ to describe the selection process.

### Description of included articles

**Figure 3.**
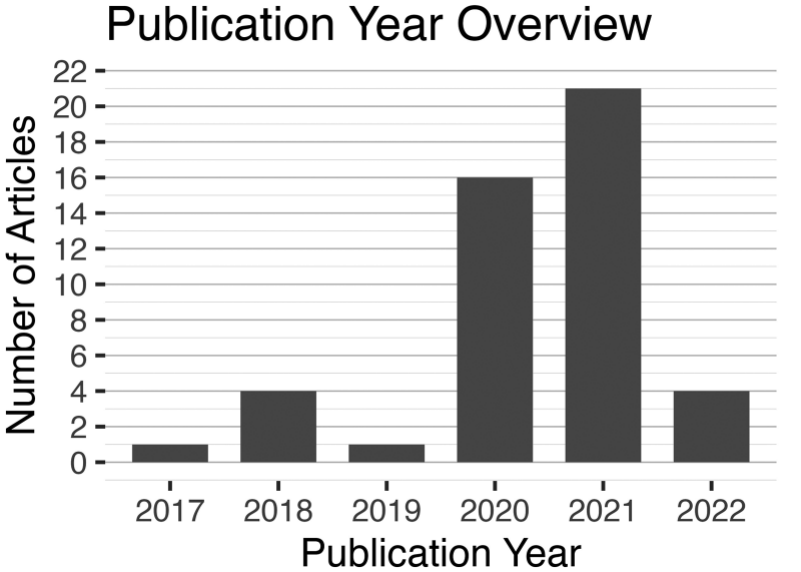
The bar plot shows the number of included articles in this review per publication year.

The following paragraph will describe the properties of the articles included in the review. Figure 3 presents the number of articles included in the review per publication year.

**Figure 4.**
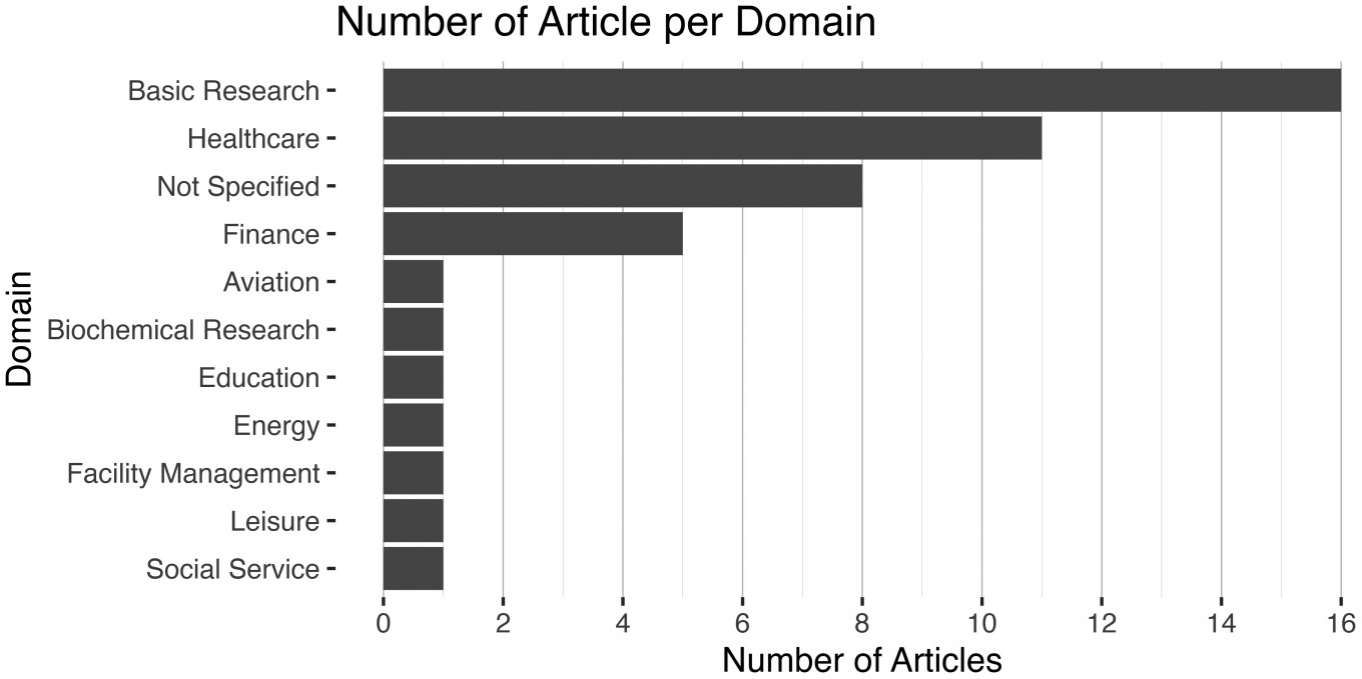
The bar plot presents the number of articles per domain.

For an overview of the number of articles per domain, see Figure 4. Most frequently the included articles belonged to the created domain of basic research (16) that emerged during the data chartering. The category contains articles from a wide variety of domains that have in common that the use case or dataset was selected based on convenience or availability or that the driving force for the research appeared not to be an application of XUIs in a specific domain but rather a general advancement of knowledge. For articles that were not categorized as belonging to the basic research domain, the most common domains were healthcare (11 articles) or the finance domain (5 articles). The remaining 14 articles did either not focus on a specific domain (8 articles; e.g., literature review) or were the only articles from a domain (7 articles).

The articles included in the review used a wide variety of methods. Many articles used multiple methods. The methods used varied depending on the domain; the most common method for articles that did not specify a domain was a literature review. Articles in the healthcare domain used the most variety of methods, with interviews being the most common and only a few experiments reported in this domain.

### Design recommendations

A total of 240 individual recommendations were extracted from the 47 articles included in the data chartering. These include eight frameworks for designing XUIs described in Table 2. Of the 240 recommendations, the authors of this review deduced 153 based on the information provided in the articles, and 79 were provided in the original articles.

**Table 2.** Framework overview.

Framework Category	Content description	Source Article
Design	The framework highlights the importance of considering “user mindset,” “user engagement,” and “knowledge outcomes” in the design of XUIs For each aspect, the authors provide organizing subcategories For all combinations of subcategories of the different aspects, the authors provide informative examples from the scientific literature in a table	A75^45^
	The framework contrasts information about “how people should reason and explain” with “how people actually reason (with errors)”; this information is linked to “how XAI generates explanations” and “how XAI supports reasoning (and mitigates errors)” This framework can be used to first identify users’ goals and associated biases, and then to consider appropriate explanations	A24^46^
	The framework highlights aspects of the context of use that may be valuable to consider when designing XUIs (Why, Who, When/Where) The framework emphasizes that these aspects guide the design of XUIs (what should be explained, how should the explanation be designed)	A435^47^
	The framework provides a list of questions that users of XUIs might ask during an interaction This framework might be used to identify user information needs for XUIs	A40^48^
Process	The framework emphasizes the interaction between the ML model, the XAI method, and the user In conjunction with the framework, the steps of the XUI design process are highlighted Domain analysis Requirements elicitation Multimodal interaction design & evaluation	A446^49^
	The framework structures the XUI design process First, the explanations for the user should be designed. Second, the models should be generated Finally, the explainability should be evaluated	A155^22^
Evaluation	The framework provides self-assessment questions that can be used for an initial evaluation of an XAI method The framework is mainly centered on the developer's point of view	A140^50^
	In this article, the authors propose a process for evaluating XUIs in the context of recommender systems, which can serve as a framework The process describes the interaction between the user and the XUI of the recommendation system, with an emphasis on the user's decision/action This description could be useful when planning evaluations of XUIs	A145^51^

The clustering of the extracted 240 individual recommendations resulted in 64 summarized basic recommendations, not counting the frameworks. Structuring these 64 recommendations revealed five questions that might be asked when designing an XUI. An individual mind map branch was created for each question, grouping the recommendations that might be useful when answering the question.

Branch 1: What information to provide in XUIs? (Summarized recommendations: 14, with 11 recommendations containing at least one healthcare article)

Branch 2: How to design XUIs? (Summarized recommendations: 29, with 17 recommendations containing at least one healthcare article)

Branch 3: How to evaluate XUIs? (Summarized recommendations: 5, with 1 recommendation containing at least one healthcare article)

Branch 4: How to configure the design process of XUIs? (Summarized recommendations: 2, with 1 recommendation containing at least one healthcare article; not counting the frameworks)

Branch 5: What to keep in mind when designing XUIs? (Summarized recommendations: 14, with 2 recommendations containing at least one healthcare article)

During the recommendation clustering process, two types of summarized recommendations emerged. The first type of recommendation was intended to provide guidance on what design decisions to make when designing XUIs. The second type reminds the reader of possible pitfalls, caveats, or design space constraints that should be considered when designing XUIs. The second type was highlighted in the mind map with a traffic cone icon and a “Be aware” at the beginning of the recommendation (e.g., H in Figure 1). The branch dealing with the question “What to keep in mind when designing XUIs” consists exclusively of the second type of recommendations. In this mind map branch, the traffic cone icon and “Be aware” were not used after the first layer to reduce redundancy.

**Figure 5.**
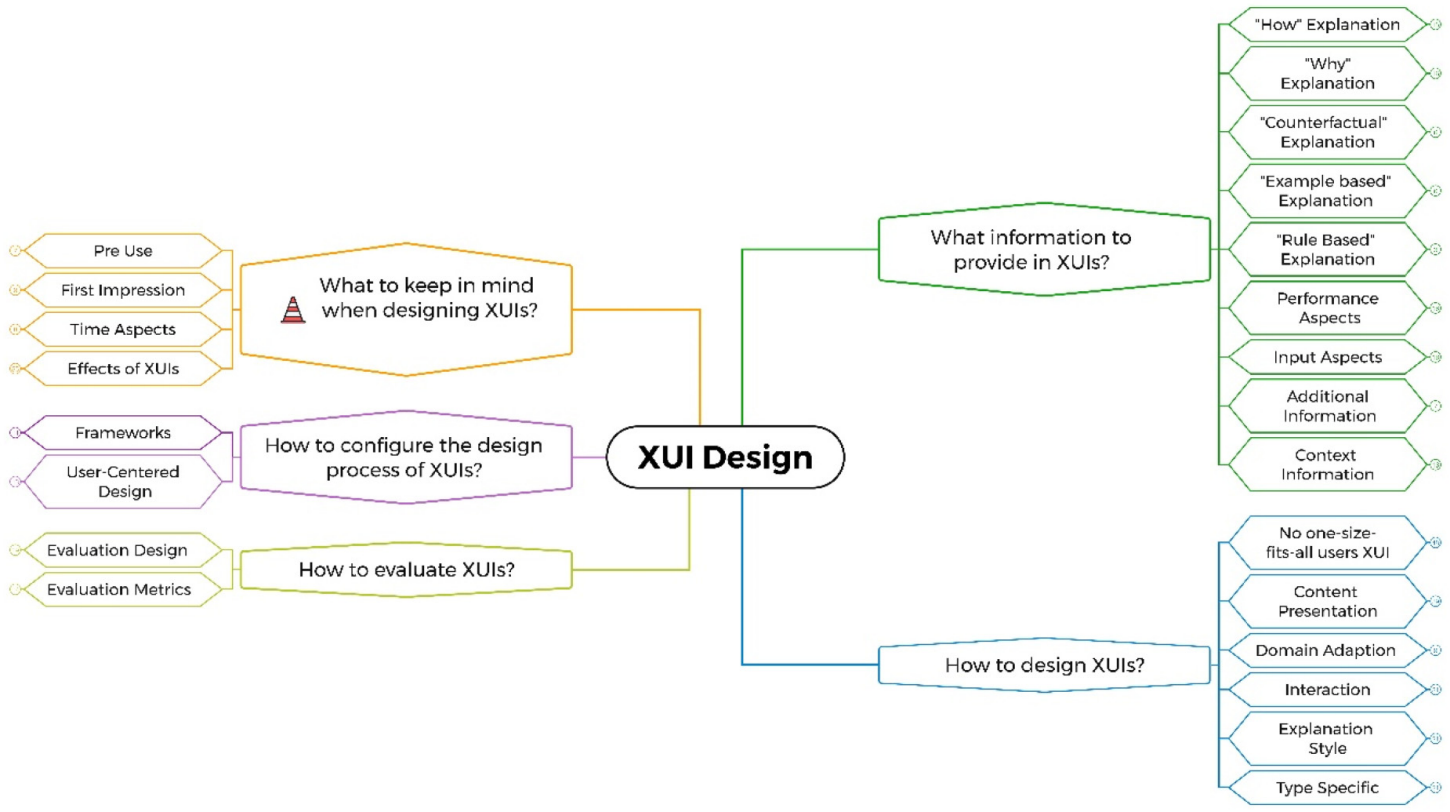
Mind map unfolded until the first layer of orientation nodes.

This mind map represents the main result of the scoping review; the illustration (Figure 5) shows only the top level of the mind map, the full mind map cannot be illustrated here due to its size, but can be found in the appendix (Semi-unfolded: Appendix G; Fully-unfolded: Appendix H).

### Design recommendations on the sublevel

**Figure 6.**
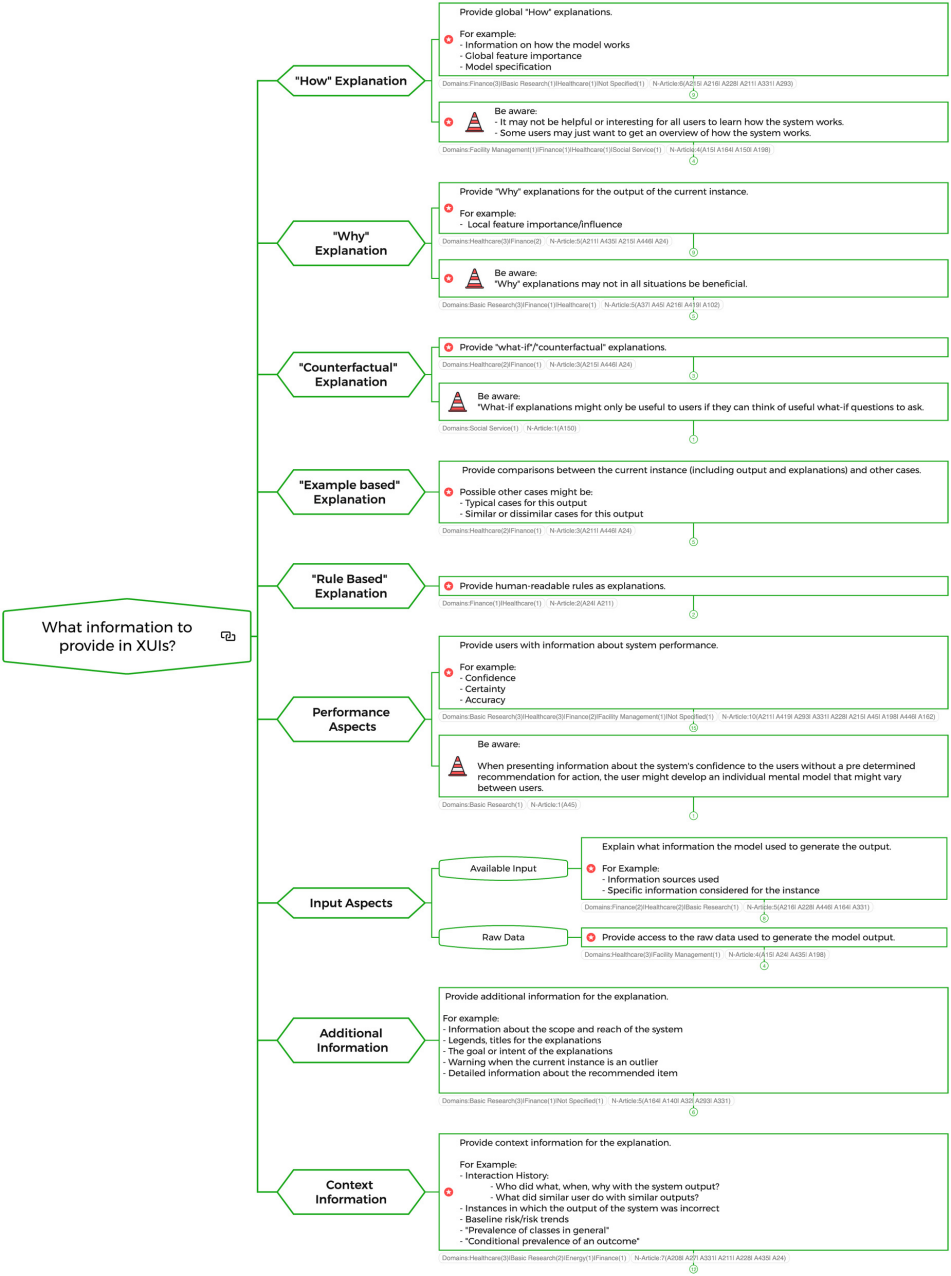
Mind map branch: “What information to provide in XUIs?”.

Following the five created mind map subbranches are broadly described textually. Not every detail is described to enhance the readability. As a visual example of the whole mind map, the first branch, “What information to provide in XUIs?” unfolds until the summarized recommendation layer is shown in Figure 6.

#### Branch 1: What information to provide in XUIs?

The first mind map branch organizes recommendations that give the designer of XUIs ideas about the information that might be valuable to present to the user in XUIs. Based on the information gathered from the included articles, it might be valuable to consider presenting certain types of recommendations in XUIs: “How”—explanation, “Why”—explanation, “Example based”—explanation and “Rule based”-explanation; information describing the performance of the AI system or information about the input aspects of the system, which may include an indication of what data were available when the system generated the output or the availability of access to the raw data. Furthermore, adding supplementary information that complements the explanations provided in the XUI might be considered. Consideration might be given to (a) providing additional information in the XUI that helps to understand the XUI and its explanation (e.g., labels or legends for the explanation itself) or (b) providing contextual information for the explanation provided in the XUI (e.g., an interaction history).

#### Branch 2: How to design XUIs?

The second branch aggregates recommendations that might be considered when designing the information that is selected to be presented in an XUI. The recommendations in this branch range from broad general recommendations to more specific recommendations for the design of certain types of explanations. For the more general recommendations, it might be considered that there are no “one size fits all” XUIs. Instead, it should be considered to adapt the XUIs to the user groups; this could be done, for example, by adapting the XUIs to the AI knowledge of the user groups. It might also be considered to personalize the explanations. Thought should be given to the presentation of the content in the XUI. What level of detail should be chosen? Should detailed information be provided on demand for interested users? How many types of explanations should be provided in an XUI? It might be thought to adapt the XUIs to the application domain's vocabulary and the users’ domain knowledge. Furthermore, should the interaction between the user and the XUI be carefully designed, with possible emphasis, for example, on the design of the interaction sequence in which the user interacts with the XUI, unrestricted access to information provided in the XUI, the ability to correct the system and easy access to the explanation in connection with the system´s output. Additionally, should the style for the explanations be chosen carefully, it might be considered to provide explanations that, for example, are engaging and informative, contain actionable information, are in an argumentative style, support the scientific process, are nontechnical explanations for end users, or evolve during the interaction history. More specific recommendations are provided for “Example based”-explanations, performance aspects, analogy explanations, and “Why”-explanations.

#### Branch 3: How to evaluate XUIs?

The third branch catalogs the recommendations for evaluating XUIs. The branch presents ideas about what to consider when designing an evaluation. For example, evaluations should be performed with users, it might be valuable to use a mix of subjective and objective measures, and control groups or conditions should be used. The branch provides a list of factors/metrics that might be evaluated for XUI in general and more specific aspects for the evaluation of analogy explanations.

#### Branch 4: How to configure the design process of XUIs?

The fourth branch compiles information relevant to the user-centered design of XUIs and organizes frameworks that may be relevant to the development of XUIs. In the “Frameworks” subbranch, frameworks are categorized according to their relevance to the design, the structuring of the design process, or the evaluation of XUIs. More detailed descriptions can be found in Table 2. For the user-centered design, the branch emphasizes an iterative design process that involves user engagement and considers the future usage context. Additionally, it highlights the communication during the design process regarding the user's mindset, the explanations’ goals, and user engagement.

#### Branch 5: What to keep in mind when designing XUIs?

The fifth branch contains recommendations that illustrate potential pitfalls when designing XUIs. The branch is intended to sensitize the readers to the potential pitfalls they may encounter when designing XUIs. Based on the literature, the designer of XUIs should be aware that before using a system with XUIs, users may already hold attitudes regarding the system and that the presence of an XUI might affect the users. Furthermore, the first impression of an XUI and the system (e.g., system errors) might influence the interaction with the system, and explanations might not be able to counterbalance this first impression. Regarding time aspects, one should be mindful that the user might have limited time to interact with the XUI, that XUIs might influence usage and reaction times, and that the users viewing time of XUIs might decrease over time. In addition, one should be aware of possible positive effects (such as increased user confidence or perceived usefulness) and negative effects (such as cognitive biases or information overload) observed for XUIs. The effects of XUIs might be moderated by, for example, metric hacking, and the correctness of the system output, the user's mental model, or the duration of use. Additionally, the user's perception of the system functionality and quality might be affected by the presence of XUIs.

### Analysis of the recommendations identified

For the most summarized recommendations, a limited number of articles built the basis, 69% of summarized recommendations are based on information from three or fewer articles. Only a few recommendations are based on a larger number of articles. Furthermore, the branches of the mind map differ in the number of articles from which the information has been extracted. The healthcare sector is represented with articles in all branches, but healthcare articles are not as frequent in the branches “How to evaluate XUIs?” and “What to keep in mind when designing XUIs?”. Half of all the summarized recommendations have at least one article as a basis that belongs to the healthcare domain. The articles categorized as belonging to the healthcare domain all describe some form of CDSS. The recommendations based on articles from the healthcare domain are therefore relevant to the design of XUIs for CDSS.

### Framework overview

Apart from the extracted recommendations, a limited number of frameworks were encountered, which might be useful to consult when designing XUIs. In the data extraction table, these are marked with a “#F” at the beginning. The frameworks are described in bullet points in Table 2. For a complete overview of the frameworks, please consult the original articles.

## Discussion

This scoping review was conducted to gather recommendations from the scientific literature for the user-centered design of XUIs to answer the research questions, “1. *What recommendations exist for a user-centered design of explanations or explanation user interfaces for AI-based systems in general?” *and* “2. What recommendations exist for the user-centered design of explanations and explanation user interfaces for AI-based decision support systems in the medical domain?”*

The review process resulted in 47 included articles. As part of the data extraction, 240 recommendations were extracted. These 240 recommendations were grouped into 64 summarized basic recommendations while creating a mind map.

Although the number of articles included in this review was limited, the information provided in the articles was sufficient to extract a large number of basic design recommendations. These recommendations deal with a wide variety of topics related to the design of XUIs: the identification of the content to present in XUIs, the design decisions to be made for the content to be displayed in the XUIs, the evaluation of XUIs, the structuring of the design process itself for XUIs and pitfalls which might be encountered when dealing with XUIs. The articles that form the basis of these recommendations have a strong presence of articles from the healthcare domain (half of the summarized recommendations are based on at least based on one healthcare article) and are augmented by articles from other domains. Based on the selected approach, a wide-reaching overview of recommendations for the design of XUIs was composed in the form of a mind map.

The level of detail of the extracted and ultimately summarized recommendations varies from specific to broad. This can be explained by the different levels of detail and content reported by the authors in the original articles from which the recommendations were extracted. As a result, the recommendations had to be written in broad terms. Therefore, the recommendations should not be seen as rigid design guidelines for all possible situations but rather as basic recommendations of what might be considered for the user-centered design of XUIs.

At this time, evaluating the evidence for the recommendations provided in a scoping review is impossible. The summarized recommendations vary in the number of articles on which they are based. More than half of the summarized recommendations are based on only 1 or 2 articles, which may indicate the field's diversity and infancy. A few recommendations are based on information from a larger number of articles. A large number of articles as a basis cannot automatically be assumed to provide a high level of evidence for a recommendation because, in some cases, the recommendations extracted from the articles are not without contradictions. The number of articles reflects the level of research interest in the aspect rather than the evidence base for a recommendation. Additionally, a wide variety of methods are deployed in the articles from which the recommendations have been extracted. It is beyond the scope of this scoping review to evaluate the investigative power of these methods. Therefore, conflicting results of articles cannot be properly assessed.

Nevertheless, this scoping review has systematically and traceably identified an extensive list of basic recommendations for the user-centered design of XUIs. These recommendations have a high degree of scientific legitimacy based on the methodology used to collect and aggregate the recommendations, the traceable manner in which the excerpts from the original literature used to create the recommendations are documented, and the inclusion criteria for the source articles. In addition, the broad wording of the summarized recommendations should not be seen as a limitation or shortcoming of the review. However, the broadness of the recommendations may be seen as strength. The broadness might make them more accessible for readers without a deep background in topics of XAI or XUI. Additionally, the broadness might make the recommendations useful for a wide range of use cases instead of very specific recommendations, which would only apply to a specific type of CDSS, in a specific context of use, and for a narrow user group. To the best of the authors’ knowledge, this is the first review that has compiled such an extensive set of basic recommendations for the complete user-centered design of XUIs rather than focusing on narrow subareas of XUI design.

### Recommendations from the health domain

As for the second research question, “*2. What recommendations exist for the user-centered design of explanations and explanation user interfaces for AI-based decision support systems in the medical domain?*” it has been observed that healthcare articles have a strong presence as the basis for the summarized recommendations. Out of the 47 articles included in the review, 11 are from the healthcare domain, and half of the summarized recommendations have at least one article from the healthcare domain as a base. All articles from the healthcare domain included in this review deal with some form of CDSS. Therefore, it can be assumed that the recommendations highlighted in the mind map as having a basis in the healthcare domain are highly relevant for other AI-based CDSS. However, we would like to point out to readers that the results of the scoping review do not replace the user-centered design process for XUIs, which ensures that XUIs meet the needs of the identified user groups in the identified context of use. The evidence base is insufficient to provide strict design guidelines for specific types of CDSS used by particular user groups in specific contexts of use. The user needs for XUI for AI-based CDSS are expected to differ between end-user groups (e.g., neurologist vs. physiotherapist), the context of use (e.g., medical specialty: emergency medicine vs. rehabilitation or work environment: doctor's office vs. emergency room), and the resulting differences in medical decision-making processes. Instead, the collected recommendations are intended to be a tool that can be consulted during the user-centered design process. This is echoed in the mind map. The call for a user-centered design process for XUIs is a recommendation extracted from the literature. The data extraction table provides information for the specific context of use described in the original paper.

A proof of efficacy (i.e., the influence on effectiveness, efficiency, satisfaction, and acceptability of use) for the recommendations in general and for recommendations without a base of articles from the healthcare domain, a proof of transferability to the healthcare is still lacking. This is important to acknowledge because the user needs and preferences in the healthcare domain might vary from those in other domains due to the high workload, time pressure, severe consequences of the decisions, need for accountability, high demand for error minimization, and safety demands experienced in the medical domain. Therefore, it should be diligently investigated whether the design recommendations from other domains hold for AI-based CDSS. However, raising awareness of thoughtful user-centered design of XUIs is a necessary step in building a foundation upon which user-centered AI-based systems in the healthcare domain can be built and adapted in the future. With its basic recommendations, the mind map is intended to provide projects in the medical informatics domain without deep knowledge of usability and XAI a starting point for their endeavors to design user-centered XUIs while sensitizing for the complexity and possible pitfalls. Additionally, by consulting the information provided in the data extraction table and the ability to identify relevant text passages in the original articles, the readers can assess the appropriateness of the recommendation for their specific situation.

### Comparison with other reviews

Rather than comparing this scoping review with the more technical reviews focusing on XUIs described in the introduction, the results of this scoping review can be best compared to a handful of reviews. In their work, Nunes and Jannach^30^ comprehensively review explanations of advice-giving systems. One result of the review was taxonomy for explanations summarizing the content of the explanations which implicitly highlights some design decisions that must be made when designing XUIs. However, Nunes and Jannach did not provide a catalog of recommendations for the design of XUIs. Additionally, this scoping review gathered information from articles that are more recent and focused on XUIs for AI-based systems than the review of Nunes and Jannach. Another relevant review is the review from Mohseni et al.,^25^ in which the authors gathered design goals for XUIs and evaluation methods. Although the review was not performed to provide an extensive overview of recommendations for the user-centered design of XUIs, does it contain seven design guidelines and a framework for an iterative design and evaluation of XAI systems. Based on the description in the article, it is, however, not clear what inclusion and exclusion criteria were used to select the articles on which the recommendations are based. This scoping review differs from the works of Mohseni et al. in several aspects, for example: in this scoping review, the inclusion and exclusion criteria are clearly described, the scoping review does not use a snowballing methodology for articles focused on the computer science literature for the literature search and the focus was to gather and organize recommendations, which allows for a different type of result presentation.

As described in the introduction, there are other reviews that focus on more specific aspects of the design of XUIs than this review. For example, after the start of this scoping review, a review focusing on identifying the information needs for a user-centered XUI design for diagnostic systems has been published.^40^ The review results can be compared with the recommendation in the first branch of the mind map. He et al. present a needs library for the medical domain for XUIs. Its content does not contradict the findings of the mind map but describes the information needs at a more detailed level. For the interested reader, the results of the review by He et al. complement the findings presented in the first branch of the mind map.

Readers interested in more information about the interaction design between the users and XUIs might consult the review by Chromik and Butz.^27^ In their review, the authors describe seven different interaction concepts and provide the reader with four recommendations for interaction design: 1. “*Consider complementing implicit explanations with rationales in natural language.*”, 2. “*Consider offering hierarchical or iterative functionalities that allow follow-ups on initial explanations.”, 3. “Consider offering multiple explanation methods and modalities to enable explainees to triangulate insights.”, and 4. “Consider offering functionalities to adjust explanations to explainees’ mental models and contexts.*”. The first recommendations could be interpreted as an addition to the recommendations in this mind map, for the point “Explanation Style” in the second mind map branch. The recommendations in the mind map reflect the following three recommendations.

With regard to the reviews focusing on the evaluation of XUIs, the review by Sperrle et al.^31^ concentrates on the human-centered evaluation of machine-learning systems. In it, the evaluation of the explanation for the ML systems focuses on four properties: transparency, trustworthiness, effectiveness, and fidelity, but the evaluation of XUIs is not the main topic of the review. The authors provide an example of a reporting format for human-centered evaluations of ML systems; this reporting format may be useful for evaluating XUIs. Additionally, the review of Zhou et al.^32^ has made the evaluation of explanations for ML a focal point; it can serve as an entry point in evaluation methods and metrics for interested readers. The review emphasizes the importance of application and human-centered evaluations that are relevant to the evaluation of XUIs. Lopes et al.^33^ provide an overview of methods for human- and computer-centered evaluation of XAI systems. For human-centered evaluation, they emphasize the importance of trust, understandability, usefulness, satisfaction, and performance.

For readers whose interest has been sparked by the description of the potential pitfalls of XUIs, it may be valuable to consult the review by Bertrand et al.^35^ on the topic of cognitive biases in XAI-based decision-making. The authors provide an overview of the biases encountered in XAI-based decision-making and present an initial list of possible mitigation strategies. Their review can be seen as a deep dive into a specific aspect of the topic: negative effects of XUIs (encountered in the fifth branch of this scoping review).

### Limitations

Besides the strength of this scoping review, the reader should consider the following limitations. No validation by external researchers or domain experts for the extracted recommendations and their summarized versions was performed, and the authors of this review deduced substantial number of the recommendations from the information reported in the included articles. To mitigate these limitations, the review followed a traceable methodology during the review process, and at a minimum, the four-eyes principle was applied at critical stages of the review. Additionally, this scoping review considers a fixed five-year period, from 2017 to 2022, which limits its comprehensive nature. The scoping review was not intended to generate a complete list of design recommendations for XUIs, which would be generated with a systematic review; instead, it was performed to showcase the available evidence at that point in time for the user-centered design of XUIs. The scoping review is proof of concept and a call to action for the field to build a regularly updated body of recommendations that represent the complete state of research for the user-centered design of XUIs based on repeated, systematic reviews. An implicit result of this scoping review is that the volume of identified research might be sufficient to warrant the effort for systematic reviews in the future when accounting for a continuously growing research effort. Furthermore, the comprehensibility of the recommendations (in terms of self-explanatory phrasing and level of detail of the wording) was not evaluated; therefore, no statement can be made as to whether the mind map is self-explanatory and can serve solely as a catalog of recommendations. However, the complete data extraction table is provided in the digital appendix. With this information, the reader can consult the exact passages in the original articles when in doubt about the content of a recommendation.

### Future work

For future research, it might be valuable to focus on aspects of XUI design for which this scoping review found few articles. By focusing on these aspects, it may be possible to replicate results and strengthen the evidence base for or against specific recommendations. Efforts could be made to test the transferability of recommendations that are not yet supported by articles from the healthcare domain to the healthcare domain. Another avenue for future research would be to investigate the effectiveness of the format in which the recommendations were presented in this scoping review to determine whether the recommendations’ type, phrasing, and structuring provide the intended benefit. Furthermore, a systematic review could be conducted to lay the foundation for official design guidelines for XUIs in the healthcare domain, with proper evaluation of the evidence base.

## Conclusion

Through this scoping review, it was possible to collect a variety of basic recommendations for the design of user-centered XUIs from the scientific literature. Although this review was not able to produce a list of generally applicable design recommendations that can be used as strict guidelines for the design of XUIs for AI-based CDSS, due to the complexity and infancy of the research topic and the limitations of a scoping review, by collecting, aggregating, and structuring the recommendations found in the current scientific literature and presenting the results in a mind map, this scoping review serves as an entry point for the design of XUIs for AI-based CDSS. It highlights design decisions that should be made during the design process and sensitize possible pitfalls when dealing with XUIs. In addition, highlighting recommendations that already have a basis from articles in the healthcare domain provides a starting point for the design of user-centered XUIs in the healthcare domain. The combination of the mind map and the data extraction table allows interested readers to assess the applicability of their use cases. The results of this scoping review emphasize the variety of aspects to be considered during the design and the complexity of the design decisions during the user-centered design of XUIs.

## Supplemental Material

sj-docx-1-dhj-10.1177_20552076241308298 - Supplemental material for Overview of basic design recommendations for user-centered explanation interfaces for AI-based clinical decision support systems: A scoping review Supplemental material, sj-docx-1-dhj-10.1177_20552076241308298 for Overview of basic design recommendations for user-centered explanation interfaces for AI-based clinical decision support systems: A scoping review by Ian-C. Jung, Katharina Schuler, Maria Zerlik, Sophia Grummt, Martin Sedlmayr and Brita Sedlmayr in DIGITAL HEALTH

sj-docx-2-dhj-10.1177_20552076241308298 - Supplemental material for Overview of basic design recommendations for user-centered explanation interfaces for AI-based clinical decision support systems: A scoping review Supplemental material, sj-docx-2-dhj-10.1177_20552076241308298 for Overview of basic design recommendations for user-centered explanation interfaces for AI-based clinical decision support systems: A scoping review by Ian-C. Jung, Katharina Schuler, Maria Zerlik, Sophia Grummt, Martin Sedlmayr and Brita Sedlmayr in DIGITAL HEALTH

sj-docx-3-dhj-10.1177_20552076241308298 - Supplemental material for Overview of basic design recommendations for user-centered explanation interfaces for AI-based clinical decision support systems: A scoping review Supplemental material, sj-docx-3-dhj-10.1177_20552076241308298 for Overview of basic design recommendations for user-centered explanation interfaces for AI-based clinical decision support systems: A scoping review by Ian-C. Jung, Katharina Schuler, Maria Zerlik, Sophia Grummt, Martin Sedlmayr and Brita Sedlmayr in DIGITAL HEALTH

sj-docx-4-dhj-10.1177_20552076241308298 - Supplemental material for Overview of basic design recommendations for user-centered explanation interfaces for AI-based clinical decision support systems: A scoping review Supplemental material, sj-docx-4-dhj-10.1177_20552076241308298 for Overview of basic design recommendations for user-centered explanation interfaces for AI-based clinical decision support systems: A scoping review by Ian-C. Jung, Katharina Schuler, Maria Zerlik, Sophia Grummt, Martin Sedlmayr and Brita Sedlmayr in DIGITAL HEALTH

sj-xlsx-5-dhj-10.1177_20552076241308298 - Supplemental material for Overview of basic design recommendations for user-centered explanation interfaces for AI-based clinical decision support systems: A scoping review Supplemental material, sj-xlsx-5-dhj-10.1177_20552076241308298 for Overview of basic design recommendations for user-centered explanation interfaces for AI-based clinical decision support systems: A scoping review by Ian-C. Jung, Katharina Schuler, Maria Zerlik, Sophia Grummt, Martin Sedlmayr and Brita Sedlmayr in DIGITAL HEALTH

sj-xlsx-6-dhj-10.1177_20552076241308298 - Supplemental material for Overview of basic design recommendations for user-centered explanation interfaces for AI-based clinical decision support systems: A scoping review Supplemental material, sj-xlsx-6-dhj-10.1177_20552076241308298 for Overview of basic design recommendations for user-centered explanation interfaces for AI-based clinical decision support systems: A scoping review by Ian-C. Jung, Katharina Schuler, Maria Zerlik, Sophia Grummt, Martin Sedlmayr and Brita Sedlmayr in DIGITAL HEALTH

sj-docx-7-dhj-10.1177_20552076241308298 - Supplemental material for Overview of basic design recommendations for user-centered explanation interfaces for AI-based clinical decision support systems: A scoping review Supplemental material, sj-docx-7-dhj-10.1177_20552076241308298 for Overview of basic design recommendations for user-centered explanation interfaces for AI-based clinical decision support systems: A scoping review by Ian-C. Jung, Katharina Schuler, Maria Zerlik, Sophia Grummt, Martin Sedlmayr and Brita Sedlmayr in DIGITAL HEALTH

sj-jpg-8-dhj-10.1177_20552076241308298 - Supplemental material for Overview of basic design recommendations for user-centered explanation interfaces for AI-based clinical decision support systems: A scoping review Supplemental material, sj-jpg-8-dhj-10.1177_20552076241308298 for Overview of basic design recommendations for user-centered explanation interfaces for AI-based clinical decision support systems: A scoping review by Ian-C. Jung, Katharina Schuler, Maria Zerlik, Sophia Grummt, Martin Sedlmayr and Brita Sedlmayr in DIGITAL HEALTH

## References

[1] Kaul V Enslin S Gross SA . History of artificial intelligence in medicine. Gastrointest Endosc 2020; 92: 807–812. 32565184 10.1016/j.gie.2020.06.040

[2] Guo Y Hao Z Zhao S , et al. Artificial intelligence in health care: bibliometric analysis. J Med Internet Res 2020; 22: e18228. 10.2196/18228 PMC7424481 32723713

[3] Meskó B Görög M . A short guide for medical professionals in the era of artificial intelligence. NPJ Digit Med 2020; 3: 1–8. 33043150 10.1038/s41746-020-00333-z PMC7518439

[4] Secinaro S Calandra D Secinaro A , et al. The role of artificial intelligence in healthcare: a structured literature review. BMC Med Inform Decis Mak 2021; 21: 1–23. 33836752 10.1186/s12911-021-01488-9 PMC8035061

[5] Rajpurkar P Chen E Banerjee O , et al. AI in health and medicine. Nat Med 2022; 28: 31–38. 35058619 10.1038/s41591-021-01614-0

[6] van Leeuwen KG Schalekamp S Rutten MJCM , et al. Artificial intelligence in radiology: 100 commercially available products and their scientific evidence. Eur Radiol 2021; 31: 3797–3804. 33856519 10.1007/s00330-021-07892-z PMC8128724

[7] van Leeuwen KG de Rooij M Schalekamp S , et al. How does artificial intelligence in radiology improve efficiency and health outcomes? Pediatr Radiol 2022; 52: 2087–2093. 34117522 10.1007/s00247-021-05114-8 PMC9537124

[8] Chen M Zhang B Cai Z , et al. Acceptance of clinical artificial intelligence among physicians and medical students: a systematic review with cross-sectional survey. Front Med 2022; 9: 990604. 10.3389/fmed.2022.990604 PMC9472134 36117979

[9] Briganti G Le Moine O . Artificial intelligence in medicine: today and tomorrow. Front Med 2020; 7: 509744. 10.3389/fmed.2020.00027 PMC7012990 32118012

[10] Hua D Petrina N Young N , et al. Understanding the factors influencing acceptability of AI in medical imaging domains among healthcare professionals: a scoping review. Artif Intell Med 2024; 147: 102698. 38184343 10.1016/j.artmed.2023.102698

[11] Asan O Bayrak AE Choudhury A . Artificial intelligence and human trust in healthcare: focus on clinicians. J Med Internet Res 2020; 22: e15154. 10.2196/15154 PMC7334754 32558657

[12] Lambert SI Madi M Sopka S , et al. An integrative review on the acceptance of artificial intelligence among healthcare professionals in hospitals. NPJ Digit Med 2023; 6: 1–14. 37301946 10.1038/s41746-023-00852-5 PMC10257646

[13] Amann J Blasimme A Vayena E , et al. Explainability for artificial intelligence in healthcare: a multidisciplinary perspective. BMC Med Inform Decis Mak 2020; 20: 1–9. 33256715 10.1186/s12911-020-01332-6 PMC7706019

[14] Mohanty A Mishra S . A comprehensive study of explainable artificial intelligence in healthcare. Stud Comput Intell 2022; 1024: 475–502.

[15] Allgaier J Mulansky L Draelos RL , et al. How does the model make predictions? A systematic literature review on the explainability power of machine learning in healthcare. Artif Intell Med 2023; 143: 102616. 37673561 10.1016/j.artmed.2023.102616

[16] Cirqueira D Nedbal D Helfert M , et al. Scenario-based requirements elicitation for user-centric explainable AI: a case in fraud detection. Cham: Springer International Publishing, 2020.

[17] Markus AF Kors JA Rijnbeek PR . The role of explainability in creating trustworthy artificial intelligence for health care : a comprehensive survey of the terminology, design choices, and evaluation strategies. J Biomed Inform 2021; 113: 103655. 33309898 10.1016/j.jbi.2020.103655

[18] Gupta J Seeja KR . A comparative study and systematic analysis of XAI models and their applications in healthcare. Arch Comput Methods Eng 2024; 31: 3977–4002.

[19] Loh HW Ooi CP Seoni S , et al. Application of explainable artificial intelligence for healthcare: a systematic review of the last decade (2011–2022). Comput Methods Programs Biomed 2022; 226: 107161. 36228495 10.1016/j.cmpb.2022.107161

[20] Schwalbe G Finzel B Schwalbe G , et al. A comprehensive taxonomy for explainable artificial intelligence: a systematic survey of surveys on methods and concepts. Data Min Knowl Discov 2023; 2023: 1–59.

[21] Barredo Arrieta A Díaz-Rodríguez N Del Ser J , et al. Explainable artificial intelligence (XAI): concepts, taxonomies, opportunities and challenges toward responsible AI. Inf Fusion 2020; 58: 82–115.

[22] Vilone G Longo L . Notions of explainability and evaluation approaches for explainable artificial intelligence. Inf Fusion 2021; 76: 89–106.

[23] Adadi A Berrada M . Peeking inside the black-box : a survey on explainable artificial intelligence (XAI). IEEE Access 2018; 6: 52138–52160.

[24] Bellucci M Delestre N Malandain N , et al. Towards a terminology for a fully contextualized XAI. Procedia Comput Sci 2021; 192: 241–250.

[25] Mohseni S Zarei N Ragan ED . A multidisciplinary survey and framework for design and evaluation of explainable AI systems. ACM Trans Interact Intell Syst 2021; 11: 1–45.

[26] Longo L Brcic M Cabitza F , et al. Explainable artificial intelligence (XAI) 2.0: a manifesto of open challenges and interdisciplinary research directions. Inf Fusion 2024; 106: 102301.

[27] Chromik M Butz A . Human-XAI interaction: a review and design principles for explanation user interfaces. In: Aridto C (ed.) Human-computer interaction – INTERACT 2021. Springer International Publishing, 2021, pp.619–640.

[28] Combi C Amico B Bellazzi R , et al. A manifesto on explainability for artificial intelligence in medicine. Artif Intell Med 2022; 133: 102423. 36328669 10.1016/j.artmed.2022.102423

[29] Rubinger L Gazendam A Ekhtiari S , et al. Machine learning and artificial intelligence in research and healthcare. Injury 2023; 54: S69–S73. 10.1016/j.injury.2022.01.046 35135685

[30] Nunes I Jannach D . A systematic review and taxonomy of explanations in decision support and recommender systems. User Model User-Adapt Interact 2017; 27: 393–444.

[31] Sperrle F El-Assady M Guo G , et al. A survey of human-centered evaluations in human-centered machine learning. Comput Graph Forum 2021; 40: 543–568.

[32] Zhou J Gandomi AH Chen F , et al. Evaluating the quality of machine learning explanations: a survey on methods and metrics. Electron 2021; 10: 1–19.

[33] Lopes P Silva E Braga C , et al. XAI systems evaluation: a review of human and computer-centred methods. Appl Sci 2022; 12: 9423.

[34] Naiseh M Jiang N Ma J , et al. Explainable recommendations in intelligent systems: delivery methods, modalities and risks. In: Lecture Notes in Business Information Processing 2020; 385: 212–228.

[35] Bertrand A Belloum R Eagan JR , et al. How cognitive biases affect XAI-assisted decision-making. In: Proceedings of the 2022 AAAI/ACM conference on AI, ethics, and society. New York, NY, USA: ACM, 2022, pp.78–91.

[36] Chakrobartty S El-Gayar O . Explainable artificial intelligence in the medical domain: a systematic review. In: AMCIS 2021 Proceedings. 1., https://aisel.aisnet.org/amcis2021/art_intel_sem_tech_intelligent_systems/art_intel_sem_tech_intelligent_systems/1 (2021).

[37] Tjoa E Guan C . A survey on explainable artificial intelligence. IEEE Trans Neural Netw Learn Syst 2020; 32: 4793–4813. 10.1109/TNNLS.2020.3027314 33079674

[38] Nazar M Alam MM Yafi DE , et al. A systematic review of human-computer interaction and explainable artificial intelligence in healthcare with artificial intelligence techniques. IEEE Access 2021; 9: 1–1.

[39] Antoniadi AM Du Y Guendouz Y , et al. Current challenges and future opportunities for XAI in machine learning-based clinical decision support systems: a systematic review. Appl Sci 2021; 11: 5088.

[40] He X Hong Y Zheng X , et al. What are the Users’ needs? Design of a user-centered explainable artificial intelligence diagnostic system. Int J Human–Computer Interact 2023; 39: 1519–1542.

[41] von Elm E Schreiber G Haupt CC . Methodische anleitung für scoping reviews (JBI-methodologie). Z Evid Fortbild Qual Gesundhwes 2019; 143: 1–7. 31296451 10.1016/j.zefq.2019.05.004

[42] Tricco AC Lillie E Zarin W , et al. PRISMA Extension for scoping reviews (PRISMA-ScR): checklist and explanation. Ann Intern Med 2018; 169: 467–473. 30178033 10.7326/M18-0850

[43] Wickham H Averick M Bryan J , et al. Welcome to the tidyverse. J Open Source Softw 2019; 4: 1686.

[44] Page MJ McKenzie JE Bossuyt PM , et al. The PRISMA 2020 statement: an updated guideline for reporting systematic reviews. Int J Surg 2021; 88: 105906. 33789826 10.1016/j.ijsu.2021.105906

[45] Eiband M Buschek D Hussmann H . How to support users in understanding intelligent systems? Structuring the discussion. In: 26th International conference on intelligent user interfaces. New York, NY, USA: Association for Computing Machinery, 2021, pp.120–132.

[46] Wang D Yang Q Abdul A , et al. Designing theory-driven user-centric explainable AI. In: Association for Computing Machinery, pp.1–15.

[47] Barda AJ Horvat CM Hochheiser H . A qualitative research framework for the design of user-centered displays of explanations for machine learning model predictions in healthcare. BMC Med Inform Decis Mak 2020; 20: 1–16. 33032582 10.1186/s12911-020-01276-x PMC7545557

[48] Liao QV Gruen D Miller S . Questioning the AI: informing design practices for explainable AI user experiences. In: Proceedings of the 2020 CHI conference on human factors in computing systems. New York, NY, USA: Association for Computing Machinery, 2020, pp.1–15.

[49] Schoonderwoerd TAJ Jorritsma W Neerincx MA , et al. Human-centered XAI: developing design patterns for explanations of clinical decision support systems. Int J Hum Comput Stud 2021; 154: 102684.

[50] Dieber J Kirrane S . A novel model usability evaluation framework (MUsE) for explainable artificial intelligence. Inf Fusion 2022; 81: 143–153.

[51] Vultureanu-Albisi A Badica C Vultureanu-Albişi A , et al. A survey on effects of adding explanations to recommender systems. Concurr Comput Pract Exp 2022; 34: 01–15.

