# Supplementary material for: Overview of basic design recommendations for user-centered explanation interfaces for AI-based clinical decision support systems: A scoping review
Source: Digit Health. 2025 Jan 23;11:20552076241308298. doi: 10.1177/20552076241308298 (PMC11758527; doi:10.1177/20552076241308298)
Supplement: sj-docx-2-dhj-10.1177_20552076241308298 - Supplemental material for Overview of basic design recommendations for user-centered explanation interfaces for AI-based clinical decision support systems: A scoping review [file sj-docx-2-dhj-10.1177_20552076241308298.docx]

# UCXAI – Scoping Review Protocol

Table of Contents

[UCXAI – Scoping Review Protocol 1](#_Toc154320548)

[Research Question 1](#_Toc154320549)

[Eligibility Criteria: 1](#_Toc154320550)

[Inclusion criteria: 1](#_Toc154320551)

[Exclusion criteria: 3](#_Toc154320552)

[Information Sources 4](#_Toc154320553)

[Search 4](#_Toc154320554)

[General Search String: 4](#_Toc154320555)

[Data Base specific search Strings: 4](#_Toc154320556)

[Source Selection Process: 7](#_Toc154320557)

[Step 1: Duplicate Removal 7](#_Toc154320558)

[Step 2: Title - Abstract – Keyword Screening 7](#_Toc154320559)

[Step 3: Full-Text Scan 7](#_Toc154320560)

[Data Chartering 7](#_Toc154320561)

[Chartering Process 7](#_Toc154320562)

[Data Items 8](#_Toc154320563)

[Critical Appraisal 8](#_Toc154320564)

## Research Question

1. What recommendations exist for a user-centered design of explanations or explanation user interfaces for AI-based systems?

2. What recommendations exist for the user-centered design of explanations or explanation user interfaces for AI-based CDSS?

## Eligibility Criteria:

### Inclusion criteria:

- The article was published in the last five years (2017 to 2022).
- The article is written in English or German.
- The article describes original research or any literature review/survey.
- The article focuses on the user-centered design of explanations or explanation user interfaces for AI-based systems.
  - - Articles are considered to focus on the **user-centered design** of explanations or explanation user interfaces when:

a user-centered design process or parts of the process are described, including e.g., experiments regarding the design of explanations or explanation user interfaces

OR

recommendations for the user-centered design of explanations or explanation user interfaces are provided or are deducible from the description of the research.

- - - Articles are considered to focus on explanations or explanation user interfaces for **AI-based systems** when the explanations or explanation user interfaces are designed for:

intelligent systems

OR

artificial intelligence (AI) based systems

OR

recommender systems

OR

intelligent agents.

- The intended recipients of the explanations and the users of the explanation user interfaces are domain experts or end-users.
- The explanations and explanation user interfaces are intended to be presented to the recipients/end-users in the form of a graphical user interface.

Additional inclusion criteria for **the full-text scan**:

- The researchers have full-text access to the article at the time of conducting the scoping review.
- In the article, recommendations regarding the design of explanations or explanation user interfaces for AI-based systems are provided or are deducible from the information provided in the article.

For this scoping review, it is assumed that recommendations are deducible from the reported research if:

(

- - Design variants of the explanations or explanation user interfaces are reproducible based on the description

OR

- - The user-centered design process is reproducible based on the description of the research

)

AND

- Results of the evaluation of usability aspects (*effectiveness, efficiency or satisfaction*) of the design variants of the explanations or explanation user interfaces or the user-centered design process are reported.

AND

- The results reported in the article are sufficient to assess aspects of the usability (*effectiveness, efficiency, satisfaction)* of the design variants of the explanations or explanation user interfaces or the user-centered design process.

Positive examples of deducible recommendations are provided below in the section: Example Recommendation.

### Exclusion criteria:

- The article was published before 2017.
- The article was not written in English or German.
- The article is not an original research article or is not any form of literature review/literature survey. The article is a scientific thesis (e.g., bachelor thesis, master thesis, PhD thesis, …).
- The article focuses on backend aspects of explanation user interfaces.

The article is considered to focus on **backend aspects of explanation** **user interfaces**, when only algorithms to generate explanations for AI models, their technical capabilities or performance or aspects of data sets are discussed **or** the article focuses on preliminary work required for the development of corresponding algorithms.

- The article focuses on philosophical, legal, or ethical aspects of explainable AI.
- The explanations or explanation user interfaces are intended to be used for or presented in the form of virtual reality, augmented reality, wearables^,^ or a multimodal interface without a graphical user interface component.
- The explanations or explanation user interfaces are intended for autonomous systems.
- The intended recipients of the explanations or the intended users of the explanation user interfaces have a deep understanding of AI models (e.g., AI researchers, data scientists, data engineers, programmers, etc.)

Additional exclusion criteria for the **full-text scan**:

- The researchers have no full-text access to the article at the time of conducting the scoping review.
- The article neither provides recommendations for the design or the user-centered design process of explanations or explanation user interfaces for AI-based systems nor are recommendations for the design or the user-centered design process of explanations or explanation user interfaces deducible from the reported research of the article.

For this scoping review, it is assumed that recommendations are not deducible from the reported research if:

- Design variants of the explanations or explanation user interfaces are not reproducible based on the description of the research

Or

- The user-centered design process is not reproducible based on the description of the research

Or

- No results are reported regarding the evaluation of aspects of the usability (effectiveness, efficiency, satisfaction) of the design variants of the explanations or explanation user interfaces or the user-centered design process. Or the results were inconclusive.

Or

- The results reported in the article are not sufficient to assess aspects of the usability (effectiveness, efficiency, satisfaction) of the design variants of the explanations or explanation user interfaces or the user-centered design process.

Examples of statements from which no recommendation is deducible are provided below in the section: Example Recommendation.

## Information Sources

- Scopus
- Web of Science
- IEEE Explore
- PubMed
- ACM Digital Library
- PsychInfo

## Search

Search fields:

- Title
- Abstract
- Keywords

### General Search String:

*(Explainab* OR "Explanation" OR Transparen* OR Interpretab* OR Understandab* OR Justification)  AND (AI OR "Artificial Intelligence" OR "Machine Learning" OR ML OR "Intelligent System" OR "Intelligent Agent" OR "Recommender System") AND ("Human-Centered" OR "User-Centered" OR "Usability")*

### Data Base specific search Strings:

| Data Base | Search / Search String |
| --- | --- |
| ACM Digital Library | "query": { (Title:(Explainab*) OR Title:( "Explanation") OR Title:(Transparen*) OR Title:(Interpretab*) OR Title:( Understandab*) OR Title:(Justification) OR Abstract:(Explainab*) OR Abstract:( "Explanation") OR Abstract:(Transparen*) OR Abstract:(Interpretab*) OR Abstract:(Understandab*) OR Abstract:(Justification) OR Keyword:(Explainab*) OR Keyword:( "Explanation") OR Keyword:(Transparen*) OR Keyword:(Interpretab*) OR Keyword:(Understandab*) OR Keyword:(Justification)) AND (Title:(AI) OR Title:("Artificial Intelligence") OR Title:("Machine Learning") OR Title:(ML) OR Title:( "Intelligent System") OR Title:("Intelligent Agent") OR Title:("Recommender System") OR Abstract:(AI) OR Abstract:("Artificial Intelligence") OR Abstract:("Machine Learning") OR Abstract:(ML) OR Abstract:( "Intelligent System") OR Abstract:("Intelligent Agent") OR Abstract:("Recommender System") OR Keyword:(AI) OR Keyword:("Artificial Intelligence") OR Keyword:("Machine Learning") OR Keyword:(ML) OR Keyword:( "Intelligent System") OR Keyword:("Intelligent Agent") OR Keyword:("Recommender System")) AND (Title:("Human-Centered") OR Title:("User-Centered") OR Title:("Usability") OR Abstract:("Human-Centered") OR Abstract:("User-Centered") OR Abstract:("Usability") OR Keyword:("Human-Centered") OR Keyword:("User-Centered") OR Keyword:("Usability")) }  "filter": { Publication Date: (01/01/2017 TO 12/31/2022) } |
| Web of Science | (TI=(Explainab*) OR TI=("Explanation") OR TI=(Transparen*) OR TI=(Interpretab*) OR TI=(Understandab*) OR TI=(Justification) OR AB=(Explainab*) OR AB=("Explanation") OR AB=(Transparen*) OR AB=(Interpretab*) OR AB=(Understandab*) OR AB=(Justification) OR AK=(Explainab*) OR AK=("Explanation") OR AK=(Transparen*) OR AK=(Interpretab*) OR AK=(Understandab*) OR AK=(Justification))  AND  (TI=(AI) OR TI=("Artificial Intelligence") OR TI=("Machine Learning") OR TI=(ML) OR TI=("Intelligent System") OR TI=("Intelligent Agent") OR TI=("Recommender System") OR AB=(AI) OR AB=("Artificial Intelligence") OR AB=("Machine Learning") OR AB=(ML) OR AB=("Intelligent System") OR AB=("Intelligent Agent") OR AB=("Recommender System") OR AK=(AI) OR AK=("Artificial Intelligence") OR AK=("Machine Learning") OR AK=(ML) OR AK=("Intelligent System") OR AK=("Intelligent Agent") OR AK=("Recommender System"))  AND  (TI=("Human-Centered") OR TI=("User-Centered") OR TI=("Usability") OR AB=("Human-Centered") OR AB=("User-Centered") OR AB=("Usability") OR AK=("Human-Centered") OR AK=("User-Centered") OR AK=("Usability")) |
| Scopus | TITLE-ABS-KEY ( ( explainab*  OR  "Explanation"  OR  transparen*  OR  interpretab*  OR  understandab*  OR  justification )  AND  ( ai  OR  "Artificial Intelligence"  OR  "Machine Learning"  OR  ml  OR  "Intelligent System"  OR  "Intelligent Agent"  OR  "Recommender System" )  AND  ( "Human-Centered"  OR  "User-Centered"  OR  "Usability" ) )  AND  ( LIMIT-TO ( PUBYEAR ,  2022 )  OR  LIMIT-TO ( PUBYEAR ,  2021 )  OR  LIMIT-TO ( PUBYEAR ,  2020 )  OR  LIMIT-TO ( PUBYEAR ,  2019 )  OR  LIMIT-TO ( PUBYEAR ,  2018 )  OR  LIMIT-TO ( PUBYEAR ,  2017 ) )  AND  ( LIMIT-TO ( LANGUAGE ,  "English" ) ) |
| IEEE Explore | (Explainab* OR "Explanation" OR Transparen* OR Interpretab* OR Understandab* OR Justification)  AND  (AI OR "Artificial Intelligence" OR "Machine Learning" OR ML OR "Intelligent System" OR "Intelligent Agent" OR "Recommender System")  AND  ("Human-Centered" OR "User-Centered" OR "Usability") |
| Pubmed | ("explainab*"[Title/Abstract] OR "explanation"[Title/Abstract] OR "transparen*"[Title/Abstract] OR "interpretab*"[Title/Abstract] OR "understandab*"[Title/Abstract] OR "Justification"[Title/Abstract]) AND ("AI"[Title/Abstract] OR "Artificial Intelligence"[Title/Abstract] OR "Machine Learning"[Title/Abstract] OR "ML"[Title/Abstract] OR "Intelligent System"[Title/Abstract] OR "Intelligent Agent"[Title/Abstract] OR "Recommender System"[Title/Abstract]) AND ("Human-Centered"[Title/Abstract] OR "User-Centered"[Title/Abstract] OR "Usability"[Title/Abstract]) AND 2017/01/01:2022/12/31[Date - Publication] |
| Psychinfo | (TI Explainab* OR TI "Explanation" OR TI Transparen* OR TI Interpretab* OR TI Understandab* OR TI Justification OR AB Explainab* OR AB "Explanation" OR AB Transparen* OR AB Interpretab* OR AB Understandab* OR AB Justification OR KW Explainab* OR KW "Explanation" OR KW Transparen* OR KW Interpretab* OR KW Understandab* OR KW Justification)  AND  (TI "AI" OR TI "Artificial Intelligence" OR TI "Machine Learning" OR TI "ML" OR TI "Intelligent System" OR TI "Intelligent Agent" OR TI "Recommender System" OR AB "AI" OR AB "Artificial Intelligence" OR AB "Machine Learning" OR AB "ML" OR AB "Intelligent System" OR AB "Intelligent Agent" OR AB "Recommender System" OR KW "AI" OR KW "Artificial Intelligence" OR KW "Machine Learning" OR KW "ML" OR KW "Intelligent System" OR KW "Intelligent Agent" OR KW "Recommender System")  AND  (TI "Human-Centered" OR TI "User-Centered" OR TI "Usability" OR AB "Human-Centered" OR AB "User-Centered" OR AB "Usability" OR KW "Human-Centered" OR KW "User-Centered" OR KW "Usability") |

## Source Selection Process:

### Step 1: Duplicate Removal

- The first author removes duplicate articles after database queries

### Step 2: Title - Abstract – Keyword Screening

- Kickoff Meeting: The first author presents all involved researchers the goals of the review and the inclusion and exclusion criteria
- Calibration Phase:
  - All authors independently screen a subset of articles and discuss the results
  - The process is repeated until group understanding is reached
- Main Phase:
  - Each article is screened independently by two researchers
  - Conflicts are discussed between researchers
  - If no agreement is reached, a third researcher screens the article
    - Majority vote decides

### Step 3: Full-Text Scan

- Kickoff Meeting: The first author presents all involved researchers the goals of the review and the inclusion and exclusion criteria
- Calibration Phase:
  - All authors independently screen a subset of articles and discuss the results
  - The process is repeated until group understanding is reached
- Main Phase:
  - Each article is screened independently by two researchers
  - Conflicts are discussed between researchers
  - If no agreement is reached, a third researcher screens the article
    - Majority vote decides
  - Documentation of exclusion reasons: If the exclusion reasons provided by the researchers differed, the reason with the lowest coding number was selected.

## Data Chartering

### Chartering Process

- Kickoff Meeting: The first author presents all involved researchers the data items to be extracted
- Calibration Phase:
  - All involved researchers independently extract the data items from a subset of articles
  - Extractions are discussed in a team meeting
- Main Phase:
  - For each article, the data items are extracted independently from two researchers
  - The first author gathers the data extractions and merges the extractions in 3 rows per article in a table
    - First two rows contain the original extractions from the two researchers
    - In the third row the first author merges the content of the first two rows per article
  - Each researcher checks the merged data extractions per article
    - Disagreements are discussed and resolved between researchers

### Data Items

- Information to Identify the article
- Aim or purpose of the article
- Domain of the article
- User role of potential XUI
- Methods used in the article
- Information about an evaluation of an XUI, if one is performed
- Information about AI methods used
- Recommendations for the user-centered design of XUIs for AI-based systems

## Critical Appraisal

- The first author generates a critical appraisal tool based on the literature
- For each article, two researchers will perform the critical appraisal independently.
- The two independent scores for each article will be averaged
- Before the critical appraisal is performed, the first author presents all researchers involved the critical appraisal tool and a group discussion is held
- Data chartering and critical appraisal are performed simultaneously
