# Supplementary material for: Overview of basic design recommendations for user-centered explanation interfaces for AI-based clinical decision support systems: A scoping review
Source: Digit Health. 2025 Jan 23;11:20552076241308298. doi: 10.1177/20552076241308298 (PMC11758527; doi:10.1177/20552076241308298)
Supplement: sj-docx-3-dhj-10.1177_20552076241308298 - Supplemental material for Overview of basic design recommendations for user-centered explanation interfaces for AI-based clinical decision support systems: A scoping review [file sj-docx-3-dhj-10.1177_20552076241308298.docx]

# UCXAI – Scoping Review: Screening Handout

Table of Contents

[UCXAI – Scoping Review: Screening Handout 1](#_Toc172101140)

[Research Question 1](#_Toc172101141)

[Inclusion criteria: 1](#_Toc172101142)

[Exclusion criteria: 3](#_Toc172101143)

[Glossary 4](#_Toc172101144)

[Recommendation Example: 8](#_Toc172101145)

[Directly Provided Recommendations 8](#_Toc172101146)

[Deduced Recommendations 8](#_Toc172101147)

[Examples where no recommendations are deducible 10](#_Toc172101148)

[References: 11](#_Toc172101149)

## Research Question

1. What recommendations exist for a user-centered design of explanations or explanation user interfaces for AI-based systems?

**2.** What recommendations exist for the user-centered design of explanations and explanation user interfaces for AI-based CDSS?

## Inclusion criteria:

- The article was published in the last five years (2017 to 2022).
- The article is written in English or German.
- The article describes original research or is any literature review/ literature survey.
- The article focuses on the user-centered design of explanations or explanation user interfaces^1^ for AI-based systems.
  - - Articles are considered to focus on the **user-centered design**^2^ of explanations or explanation user interfaces when:

a user-centered design process^2^ or parts of the process are described, including e.g., experiments regarding the design of explanations or explanation user interfaces

OR

recommendations for the user-centered design of explanations or explanation user interfaces are provided or are deducible from the description of the research.

- - - Articles are considered to focus on explanations or explanation user interfaces for **AI-based systems** when the explanations or explanation user interfaces are designed for:

intelligent systems^4^

OR

artificial intelligence (AI)^3^ based systems

OR

recommender systems^6^

OR

intelligent agents^5^ .

- The intended recipients of the explanations and the users of the explanation user interfaces are domain experts^7^ or end-users^8^.
- The explanations and explanation user interfaces are intended to be presented to the recipients/end-users in the form of a graphical user interface^9^.

Additional inclusion criteria for **the full-text scan**:

- The researchers have full-text access to the article at the time of conducting the scoping review.
- In the article, recommendations regarding the design of explanations or explanation user interfaces for AI-based systems are provided or are deducible from the information provided in the article.

For this scoping review, it is assumed that recommendations are deducible from the reported research if:

(

- - Design variants of the explanations or explanation user interfaces are reproducible based on the description

OR

- - The user-centered design process is reproducible based on the description of the research

)

AND

- Results of the evaluation of usability^15^ aspects (*effectiveness, efficiency or satisfaction*) of the design variants of the explanations or explanation user interfaces or the user-centered design process are reported.

AND

- The reported results in the article are sufficient to assess aspects of the usability ^15^ (*effectiveness, efficiency, satisfaction)* of the design variants of the explanations or explanation user interfaces or the user-centered design process.

Positive examples of deducible recommendations are provided below in the section: Example Recommendation.

## Exclusion criteria:

- The article was published before 2017.
- The article was not written in English or German.
- The article is not an original research or any form of literature review/survey. The article is a scientific thesis (e.g. bachelor thesis, master thesis, PhD thesis, …).
- The article focuses on backend aspects of explanation user interfaces.

The article is considered to focus on **backend aspects of explanation** **user interfaces**, when only algorithms to generate explanations for AI models, their technical capabilities or performance or aspects of data sets are discussed **or** the article focuses on preliminary work required for the development of corresponding algorithms.

- The article focuses on philosophical, legal, or ethical aspects of explainable AI.
- The explanations or explanation user interfaces are intended to be used for or presented in the form of virtual reality^10^, augmented reality^11^, wearables^12,^ or a multimodal interface^13^ without a graphical user interface^9^ component.
- The explanations or explanation user interfaces are intended for autonomous systems^14^.
- The intended recipients of the explanations or the intended users of the explanation user interfaces have a deep understanding of AI models (e.g., AI researchers, data scientists, data engineers, programmers, etc.)

Additional exclusion criteria for the **full-text scan**:

- The researchers have no full-text access to the article at the time of conducting the scoping review.
- The article neither provides recommendations for the design or the user-centered design process of explanations or explanation user interfaces for AI-based systems nor are recommendations for the design or the user-centered design process of explanations or explanation user interfaces deducible from the reported research of the article.

For this scoping review, it is assumed that recommendations are not deducible from the reported research if:

- Design variants of the explanations or explanation user interfaces are not reproducible based on the description of the research

Or

- The user-centered design process is not reproducible based on the description of the research

Or

- No results are reported regarding the evaluation of aspects of the usability^15^ (effectiveness, efficiency, satisfaction) of the design variants of the explanations or explanation user interfaces or the user-centered design process. Or the results were inconclusive.

Or

- The results reported in the article are not sufficient to assess aspects of the usability^15^ (effectiveness, efficiency, satisfaction) of the design variants of the explanations or explanation user interfaces or the user-centered design process.

Examples of statements from which no recommendation is deducible are provided below in the section: Example Recommendation.

## Glossary

1. Explanation User Interface

An interface that displays any information that makes the output or the process of generating the output of an AI-based system more understandable to the users.

1. User-centered design process

(often used synonymously “human-centered”)

“*The User-centered design (UCD) process outlines the phases throughout a design and development life-cycle all while focusing on gaining a deep understanding of who will be using the product*.” <https://www.usability.gov/what-and-why/user-centered-design.html>
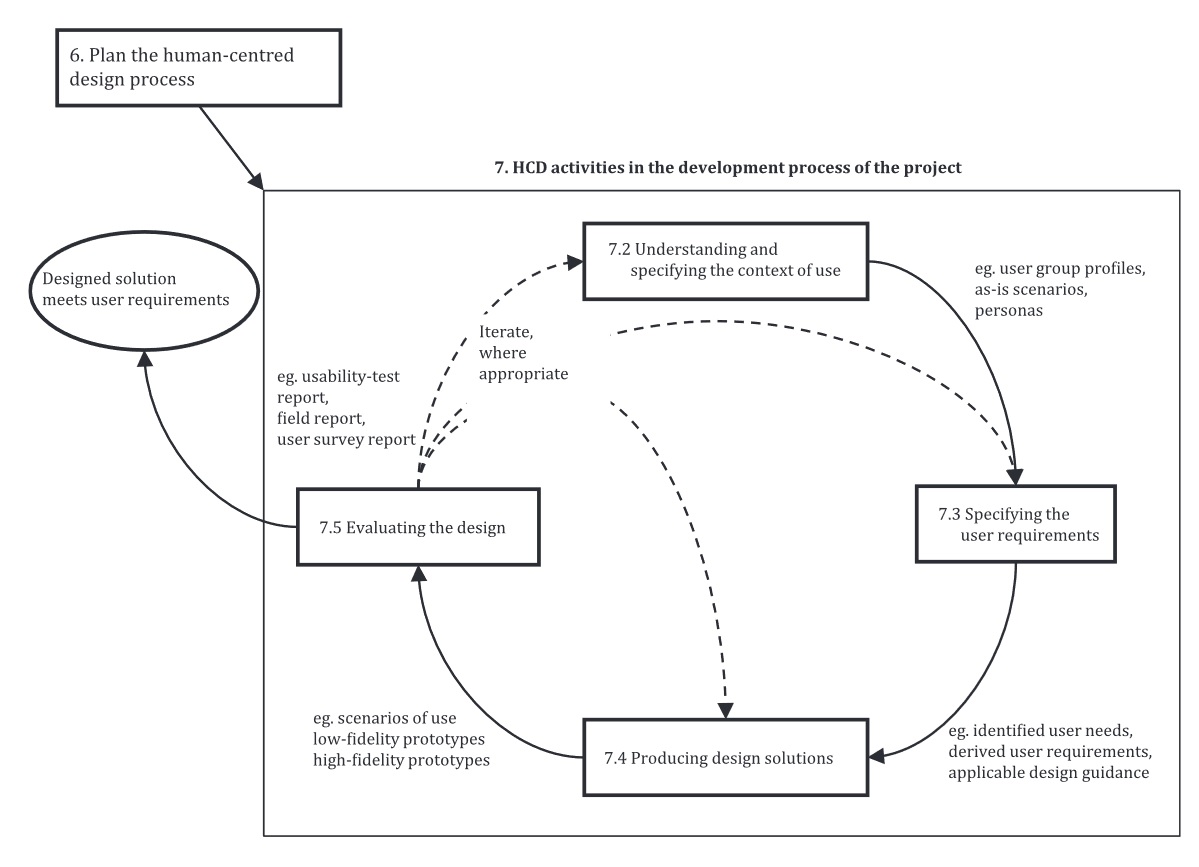


Figure 1 Human-centered design process as defined in the ISO 9421-210 (International Organization for Standardization, 2019)

1. Artificial Intelligence

For this article, the following definition of AI is used (although it is acknowledged that there is no agreed up own definition in the field of AI (Commission et al., 2020) and that what is considered as AI has changed over time (Jiang et al., 2022))

“*Artificial intelligence (AI) systems are software (and possibly also hardware) systems designed by humans that, given a complex goal, act in the physical or digital dimension by perceiving their environment through data acquisition, interpreting the collected structured or unstructured data, reasoning on the knowledge, or processing the information, derived from this data and deciding the best action(s) to take to achieve the given goal. AI systems can either use symbolic rules or learn a numeric model, and they can also adapt their behavior by analyzing how the environment is affected by their previous actions.*

*As a scientific discipline, AI includes several approaches and techniques, such as machine learning (of which deep learning and reinforcement learning are specific examples), machine reasoning (which includes planning, scheduling, knowledge representation and reasoning, search, and optimization), and robotics (which includes control, perception, sensors and actuators, as well as the integration of all other techniques into cyber -physical systems)*” (High-Level Group on Artificial Intelligence, 2019)

The following graphic shows an overview of typical methods used in AI Systems:

See (Mata et al., 2018)

A more detailed list of AI methods can be found on the following website:

https://eteppo.com/post/artificial-intelligence-simple-overview/ai-algorithms.png

1. Intelligent System

“*Intelligent System (IS) can be defined as the system that incorporates intelligence into applications being handled by machines. Intelligent systems perform search and optimization along with learning capabilities. Different types of machine learning such as supervised, unsupervised and reinforcement learning can be modeled in designing intelligent systems. Intelligent systems also perform complex automated tasks which are not possible by traditional computing paradigm. Various diagnostic, robotics and engineering systems are results of intelligent procedures implemented in Intelligent System Design.”* (Mankad, 2015)

1. Intelligent Agent

“[Intelligent agents] *are software entities that execute a series of operations to meet other software or user needs. To build them Artificial Intelligence techniques are applied.*” (Durán & Álvarez, 2015)

1. Recommender systems

“*The goal of a recommender system is to generate meaningful recommendations to a collection of users for items or products that might interest them. (…)*” (Buhmann et al., 2011)

1. Domain Expert

“*A person with special knowledge or skills in a particular area of endeavor.(…)*” (Lofaro, 2018)

1. End-user

“*the ultimate consumer of a finished product*” (Merriam-Webster, n.d.)

1. Graphical User Interface

“*An interface between a user and a computer system that makes use of input devices other than the keyboard and presentation techniques other than alphanumeric characters. Typical GUIs involve the use of windows, icons, menus, and pointing devices. The windows can contain control objects such as dialogue boxes, slider bars, radio buttons, check boxes, and pick lists, as well as textual or graphical information. The objects forming the interface display have attributes such as the ability to be resized, moved around the display, shrunk down to an icon, or given different colours. Perhaps the best-known GUIs are those used on Microsoft Windows PCs and Apple Macintosh computers, although there are several others in common use.*” (A Butterfield & Ngondi, 2016)

1. virtual reality (VR)

*“Virtual Reality is the technology that provides almost real and/or believable experiences in a synthetic or virtual way.*

*To achieve this goal, virtual reality uses the entire spectrum of current multimedia technologies such as image, video, sound and text, as well as newer and upcoming media such as e-touch, e-taste, and e-smell. (…)"* (Furht, 2008c)

1. augmented reality

“Augmented reality is a system that enhances the real world by superimposing computer-generated information on top of it. (…)” (Furht, 2008a)

1. wearables

*“Wearables are lightweight, sensor-based devices which are worn close to and/or on the surface of the skin, where they detect, analyze, and transmit information concerning several internal and/or external variables to an external device(…)*”(Düking et al., 2016)

1. multimodal interface

“*Multimodal interfaces process two or more combined user input modes, such as speech, pen, touch, manual gestures, and gaze, in a coordinated manner with multimedia system output.(…)*” (Furht, 2008b)

1. autonomous systems

There are numerous definitions of autonomy and autonomous systems (Müller et al., 2021) with different degrees of complexity. In this article, we follow Connely's and Hong's (2006) simplistic definition of autonomous systems:

“*An autonomous system is one that makes and executes a decision to achieve a goal without full, direct human control*.”

This choice was made to emphasize the importance of physicians' decision authority and responsibility while using CDSS and therefore exclude articles in which this human agency is missing.

1. Usability

Usability is defined in the ISO 9241-11 (2018) as the “*extent to which a system, product or service can be used by specified users to achieve specified goals*

*with effectiveness, efficiency and satisfaction in a specified context of use*” (International Organization for Standarization, 2018)

## Recommendation Example:

This section provides examples for statements from which recommendations can be deduced and form which no recommendations can be deduced.

### Directly Provided Recommendations

#### Real Examples

Example 1:

"*(...) enthusiastic users should be allowed to understand details, including model specifications, feature effects, and interactions."* (Deo & Sontakke, 2021, p. 47)

Example 2:

"*Essential explanations should be conveyed through interactive visualizations*”(Deo & Sontakke, 2021, p. 47)

Example 3: (Directly provided recommendation which is to long for a full quotation in the data extraction table)

Liao et al.(2020) provide a question bank of potential user questions for the interaction with XAI systems. This question bank can be used during the design process of explanation user interfaces as starting point for the identification which user question should be answered with de explanation user interface (Liao et al., 2020)

#### Hypothetical Examples

Example 4:

“*More detailed explanation texts can lead to a higher mental workload, therefore in situations in which an high mental workload can be assumed explanation texts should be held as concise as possible.*”

### Deduced Recommendations

#### Real Examples

Example 5:

**Prolog:**

Experiment with two conditions: Explanation user interface with or without feedback function.

**Statement in the article:**

"*All together, these results imply that the participants in the feedback group had greater satisfaction and understanding of how they received the recommendations than those in the non-feedback group.*" (Kim et al., 2020, p. 189019)

**Deduced recommendation:**

Explanation user interfaces in which users can provide feedback regarding the correctness of a recommendation and its corresponding explanation might lead to a higher user satisfaction and understanding than an explanation user interface without a feedback function.

Example 6:

**Prolog:**

Focus group evaluation of multiple explanation user interfaces in the healthcare domain.

**Statement in the article:**

"*Participants in our focus groups were enthusiastic about the ability to visually assess which risk factors were contributing most to an individual’s predicted risk.*" (Barda et al., 2020, p. 12)

**Deduced recommendation:**

Users of explanation user interfaces might be pleased to be presented visual presentations of the most influential features for an individual output from the model.

Example 7:

**Prolog:**

Focus group evaluation of multiple explanation user interfaces in the healthcare domain.

**Statement in the article:**

“*In particular, access to raw patient data (e.g., laboratory values, vital signs, interventions) was seen as useful for assessing the clinical credibility and utility of predictions and explanations.*” (Barda et al., 2020, p. 13)

**Deduced recommendation:**

Users of explanation user interfaces might benefit from access to the raw input data used to generate an individual output.

#### Hypothetical Examples

Example 8:

**Prolog:**

A-B-Test of two-explanation user interfaces, one with very detailed explanation texts and one with concise explanation texts, with a focus on the mental workload.

**Statement in the article:**

Explanation user interfaces with more detailed explanation texts lead to a significant higher mental workload as explanation user interfaces with more concise explanation texts.

**Deduced recommendation:**

In situations in with high mental workload explanation texts should be held as concise as possible.

Example 9:

**Prolog:**

Summative evaluation of one explanation user interface with detailed explanation texts.

**Statement in the article:**

During the evaluation of the prototype, containing the explanation user interface with a detailed explanation text, a high mental workload was observed.

**Deduced recommendation:** “*Be cautious of the users' mental workload when using detailed explanation texts in explanation user interfaces for AI-based systems.*”

### Examples where no recommendations are deducible

#### Hypothetical Examples

Example 10

**Prolog:**

A-B-Test of two-explanation user interfaces, one with very detailed explanation texts and one with concise explanation texts, with a focus on the mental workload.

**Statement in the article:**

The evaluation of the explanation user interface showed that the level of detail of the explanation text had no influence on the mental workload.

**Deduced recommendation:**

No actionable recommendation for the design of explanations or explanation user interfaces for AI-based systems is deducible.

Example 11

**Prolog:**

Different versions of explanation user interfaces were developed, and some aspects of the usability of these explanation user interfaces were evaluated. The article does not report the results of the evaluation of the different versions.

**Deduced recommendation:**

No actionable recommendation for the design of explanations or explanation user interfaces for AI-based systems is deducible based only on the description of the different explanation user interface versions.

## References:

Barda, A. J., Horvat, C. M., & Hochheiser, H. (2020). A qualitative research framework for the design of user-centered displays of explanations for machine learning model predictions in healthcare. *BMC Medical Informatics and Decision Making*, *20*(1), 1–16. https://doi.org/10.1186/s12911-020-01276-x

Buhmann, M. D., Melville, P., Sindhwani, V., Quadrianto, N., Buntine, W. L., Torgo, L., Zhang, X., Stone, P., Struyf, J., Blockeel, H., Driessens, K., Miikkulainen, R., Wiewiora, E., Peters, J., Tedrake, R., Roy, N., Morimoto, J., Flach, P. A., & Fürnkranz, J. (2011). Recommender Systems. In *Encyclopedia of Machine Learning* (pp. 829–838). Springer US. https://doi.org/10.1007/978-0-387-30164-8_705

Butterfield, A, & Ngondi, G. E. (2016). *A Dictionary of Computer Science* (Andrew Butterfield & G. E. Ngondi (Eds.)). Oxford University Press. https://doi.org/10.1093/acref/9780199688975.001.0001

Commission, E., Centre, J. R., Samoili, S., López Cobo, M., Gómez, E., De Prato, G., Martínez-Plumed, F., & Delipetrev, B. (2020). *AI watch : defining artificial intelligence : towards an operational definition and taxonomy of artificial intelligence*. Publications Office. https://doi.org/doi/10.2760/382730

Connelly, J., Hong, W. S., Mahoney, Jr., R. B., & Sparrow, D. A. (2006). Current challenges in autonomous vehicle development. *Unmanned Systems Technology VIII*, *6230*, 62300D. https://doi.org/10.1117/12.666574

Deo, S., & Sontakke, N. S. (2021). Usability, User Comprehension, and Perceptions of Explanations for Complex Decision Support Systems in Finance: A Robo-Advisory Use Case. *Computer*, *54*(10), 38–48. https://doi.org/10.1109/MC.2021.3076851

Düking, P., Hotho, A., Holmberg, H. C., Fuss, F. K., & Sperlich, B. (2016). Comparison of Non-Invasive Individual Monitoring of the Training and Health of Athletes with Commercially Available Wearable Technologies. *Frontiers in Physiology*, *7*(MAR), 71. https://doi.org/10.3389/FPHYS.2016.00071

Durán, E. B., & Álvarez, M. (2015). Ubiquitous Learning Supporting Systems: A Challenge for Computing Software Designers. In F. V Cipolla-Ficarra (Ed.), *Handbook of Research on Interactive Information Quality in Expanding Social Network Communications* (pp. 202–225). IGI Global. https://doi.org/10.4018/978-1-4666-7377-9.ch013

Furht, B. (Ed.). (2008a). Augmented Reality. In *Encyclopedia of Multimedia* (pp. 35–36). Springer US. https://doi.org/10.1007/978-0-387-78414-4_91

Furht, B. (Ed.). (2008b). Multimodal Interfaces. In *Encyclopedia of Multimedia* (pp. 651–652). Springer US. https://doi.org/10.1007/978-0-387-78414-4_159

Furht, B. (Ed.). (2008c). Virtual Reality. In *Encyclopedia of Multimedia* (pp. 968–968). Springer US. https://doi.org/10.1007/978-0-387-78414-4_255

High-Level Group on Artificial Intelligence. (2019). *A definition of AI: Main capabilities and scientific disciplines High-Level*. https://ec.europa.eu/digital-single-

International Organization for Standardization, . (2019). *Human-centred design for interactive systems (ISO standard no. 9241-210)*.

International Organization for Standarization, . (2018). *Usability : Definitions and concepts (ISO standard no. 9241-11:2018 )*.

Jiang, Y., Li, X., Luo, H., Yin, S., & Kaynak, O. (2022). Quo vadis artificial intelligence? *Discover Artificial Intelligence 2022 2:1*, *2*(1), 1–19. https://doi.org/10.1007/S44163-022-00022-8

Kim, B. H., Koh, S., Huh, S., Jo, S., & Choi, S. (2020). Improved Explanatory Efficacy on Human Affect and Workload Through Interactive Process in Artificial Intelligence. *IEEE Access*, *8*, 189013–189024. https://doi.org/10.1109/ACCESS.2020.3032056

Liao, Q. V., Gruen, D., & Miller, S. (2020). Questioning the AI: Informing Design Practices for Explainable AI User Experiences. *Proceedings of the 2020 CHI Conference on Human Factors in Computing Systems*, 1–15. https://doi.org/10.1145/3313831.3376590

Lofaro, R. J. (2018). Cognitive Ergonomics in 2016. In *Encyclopedia of Information Science and Technology, Fourth Edition* (pp. 662–670). IGI Global. https://doi.org/10.4018/978-1-5225-2255-3.ch057

Mankad, K. B. (2015). An Intelligent Process Development Using Fusion of Genetic Algorithm with Fuzzy Logic. In P. Vasant (Ed.), *Handbook of Research on Artificial Intelligence Techniques and Algorithms* (pp. 44–81). IGI Global. https://doi.org/10.4018/978-1-4666-7258-1.ch002

Mata, J., de Miguel, I., Durán, R. J., Merayo, N., Singh, S. K., Jukan, A., & Chamania, M. (2018). Artificial intelligence (AI) methods in optical networks: A comprehensive survey. *Optical Switching and Networking*, *28*, 43–57. https://doi.org/10.1016/J.OSN.2017.12.006

Merriam-Webster. (n.d.). *End user Definition & Meaning - Merriam-Webster*. Retrieved March 8, 2022, from https://www.merriam-webster.com/dictionary/end user

Müller, M., Müller, T., Ashtari Talkhestani, B., Marks, P., Jazdi, N., & Weyrich, M. (2021). Industrial autonomous systems: a survey on definitions, characteristics and abilities. *At - Automatisierungstechnik*, *69*(1), 3–13. https://doi.org/10.1515/auto-2020-0131
