# Supplementary material for: Overview of basic design recommendations for user-centered explanation interfaces for AI-based clinical decision support systems: A scoping review
Source: Digit Health. 2025 Jan 23;11:20552076241308298. doi: 10.1177/20552076241308298 (PMC11758527; doi:10.1177/20552076241308298)
Supplement: sj-docx-4-dhj-10.1177_20552076241308298 - Supplemental material for Overview of basic design recommendations for user-centered explanation interfaces for AI-based clinical decision support systems: A scoping review [file sj-docx-4-dhj-10.1177_20552076241308298.docx]

# UCXAI-Review: Exclusion Criteria Codes

| Bullet Point | Code | Description |
| --- | --- | --- |
| Wrong Publication Date | F1 | The article was published before 2017. |
| Wrong Language | F2 | The article was not written in English or German. |
| Wrong article type | E1 | The article is not an original research article or is not any form literature review/literature survey.  OR  The article is a scientific thesis (e.g. bachelor thesis, master thesis, PhD thesis, …). |
| Wrong focus: backend aspects of explanation user interface | E2 | The article focuses on backend aspects of explanation user interfaces.  The article is considered to focus on backend aspects of explainable user interfaces, when only algorithms to generate explanations for AI models, their technical capabilities or performance or aspects of data sets are discussed or the article focuses on preliminary work required for the development of corresponding algorithms. |
| Wrong focus: philosophical, legal, ethical aspects | E3 | The article focuses on philosophical or legal or ethical aspects of XAI. |
| Different Topic | E4 | The article focuses on a different topic, which has nothing to do with explanations or explanation user interfaces for AI based Systems. |
| Wrong modality | E5 | The explanations or explanation user interfaces are intended to be used for or presented in form of virtual reality, augmented reality, wearables or a multimodal interface without a graphical user interface component. |
| Autonomous System | E6 | The explanations or explanation user interfaces are intended for autonomous systems. |
| Wrong user group | E7 | The intended recipients of the explanations or the intended the users of the explanation user interfaces have a deep understanding of AI models (e.g. AI researchers, data scientists, data engineers, programmers etc.) |
| No design recommendation provided / not sufficient information for deduction | E8 * | The article neither provides recommendations for the design or the user centered design process of explanations or explanation user interfaces for AI based systems; **nor** are recommendations for the design or the user centered design process of explanations or explanation user interfaces deducible from the reported research of the article.  For this scoping review, it is assumed that recommendations are not deducible from the reported research if:   - Design variants of the explanations or explanation user interfaces are not reproducible based on the description of the research   Or   - The user-centered design process is not reproducible based on the description of the research   Or   - No results are reported regarding the evaluation of aspects of the usability**^Fehler! Verweisquelle konnte nicht gefunden werden.^** (effectiveness, efficiency, satisfaction) of the design variants of the explanations or explanation user interfaces or the user-centered design process. Or the results were inconclusive.   Or   - The reported results in the article are not sufficient to assess aspects of the usability (effectiveness, efficiency, satisfaction) of the design variants of the explanations or explanation user interfaces or the user centered design process.   Examples of statements from which no recommendation is deducible are provided in the handout under the point: Example Recommendation. |
|  |  |  |

# Inclusion due to not sufficient information:

| Bullet Point | Code | Description |
| --- | --- | --- |
| Not sufficient Information | I1 | The abstract is missing critical information for the inclusion or exclusion decision, there for the paper will be included in the full text scan |

Legend:

“*” marks exclusion criteria which are just used during the full text scan
