# Supplementary figures and images for: Overview of basic design recommendations for user-centered explanation interfaces for AI-based clinical decision support systems: A scoping review
Source: Digit Health. 2025 Jan 23;11:20552076241308298. doi: 10.1177/20552076241308298 (PMC11758527; doi:10.1177/20552076241308298)

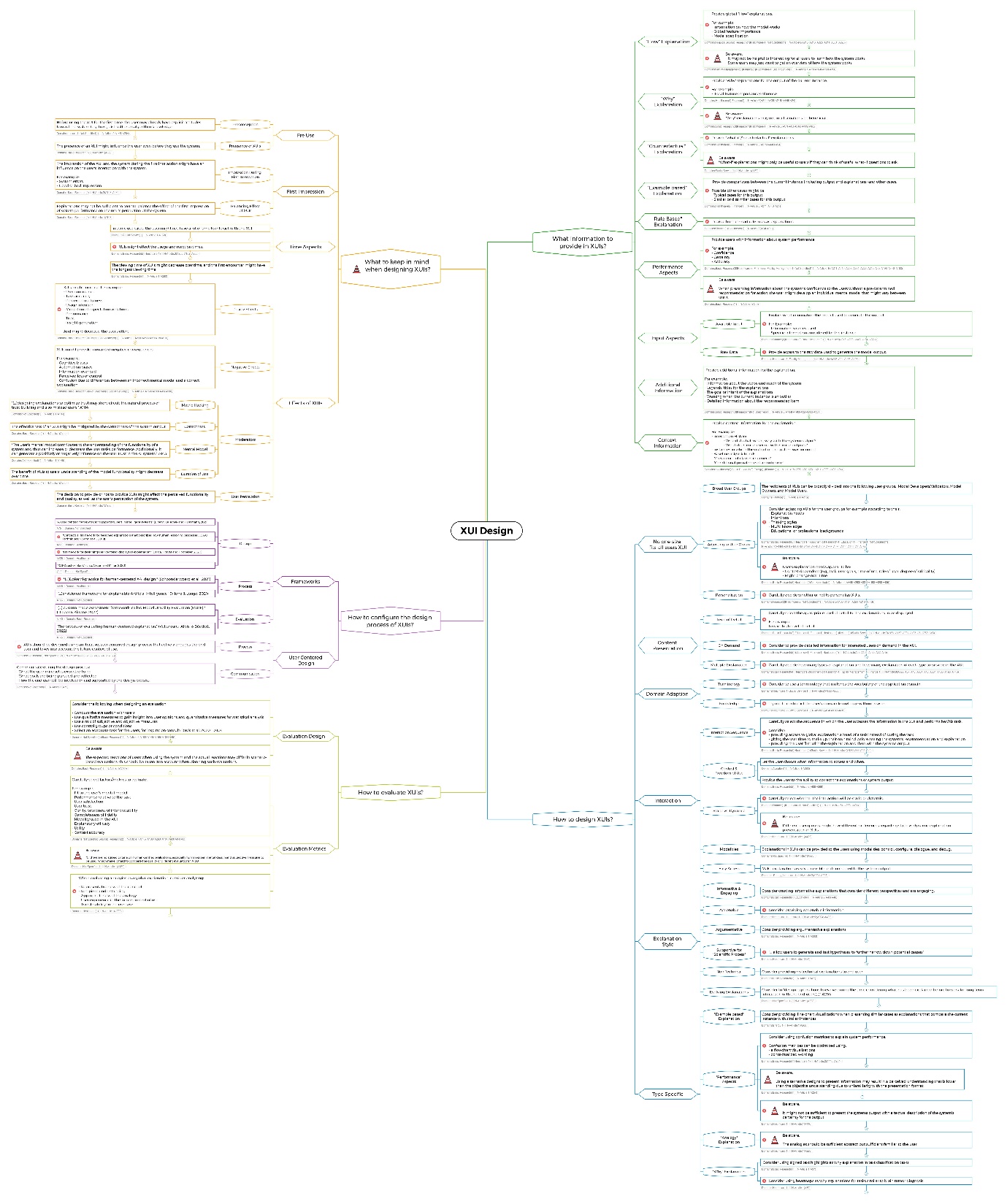

Supplement: sj-docx-7-dhj-10.1177_20552076241308298 - Supplemental material for Overview of basic design recommendations for user-centered explanation interfaces for AI-based clinical decision support systems: A scoping review [file sj-docx-7-dhj-10.1177_20552076241308298.docx]

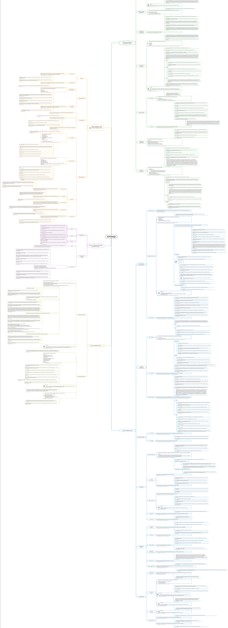

Supplement: sj-jpg-8-dhj-10.1177_20552076241308298 - Supplemental material for Overview of basic design recommendations for user-centered explanation interfaces for AI-based clinical decision support systems: A scoping review [file sj-jpg-8-dhj-10.1177_20552076241308298.jpg]
